# Supplementary material for: Unlocking the Bacterial SecY Translocon
Source: Structure. 2016 Apr 5;24(4):518–27. doi: 10.1016/j.str.2016.02.001 (PMC4826270; doi:10.1016/j.str.2016.02.001)
Supplement: Document S1. Figures S1–S7 [file mmc1.pdf]

Structure, Volume *24*

Supplemental Information

Unlocking the Bacterial SecY Translocon

Robin A. Corey, William J. Allen, Joanna Komar, Simonas Masiulis, Sam Menzies, Alice Robson, and Ian Collinson

Unlocking the bacterial SecY translocon

Robin A. Corey, William J. Allen, Joanna Komar, Simonas Masiulis, Sam Menzies, Alice Robson and Ian Collinson

Supplemental information

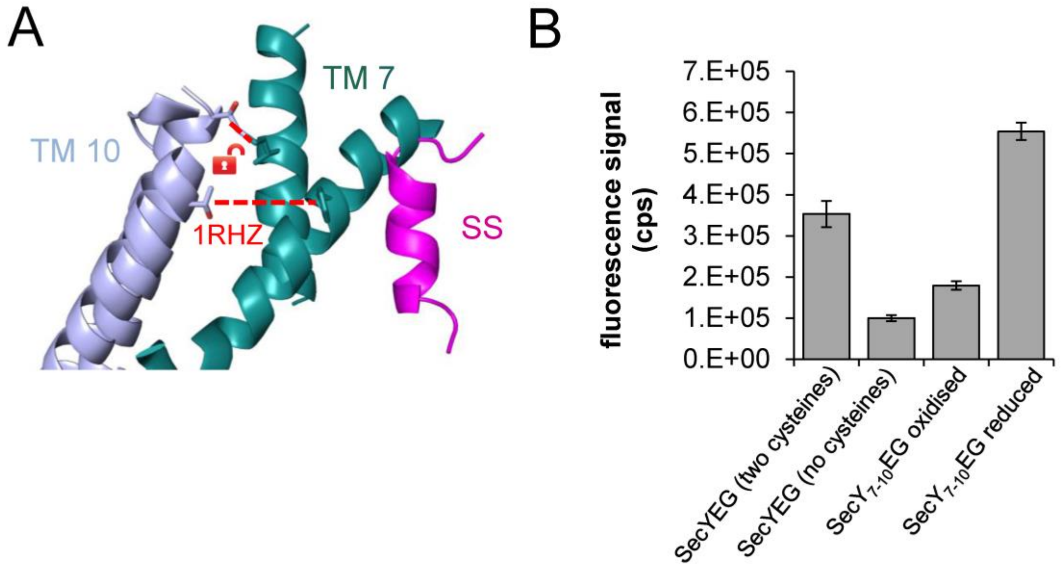

Figure S1. Related to Figure 1.

(A) Close up of SecY TMs 7 and 10 from 1RHZ (Van den Berg et al., 2004) and SS-bound SecYEG (open red padlock (Hizlan et al., 2012)) represented as cartoons and overlaid. The cysteine pair in $SecY_{7\text{-}10}EG$ are shown as sticks to compare their distances (red dashed lines) in the resting state and upon SS activation respectively.

(B) Fluorescence emission following incubation of SecYEG with the CPM dye. Fluorescence is relative to number of free thiols present and is measured in counts per second (cps). Error bars are S.E.M of three repeats.

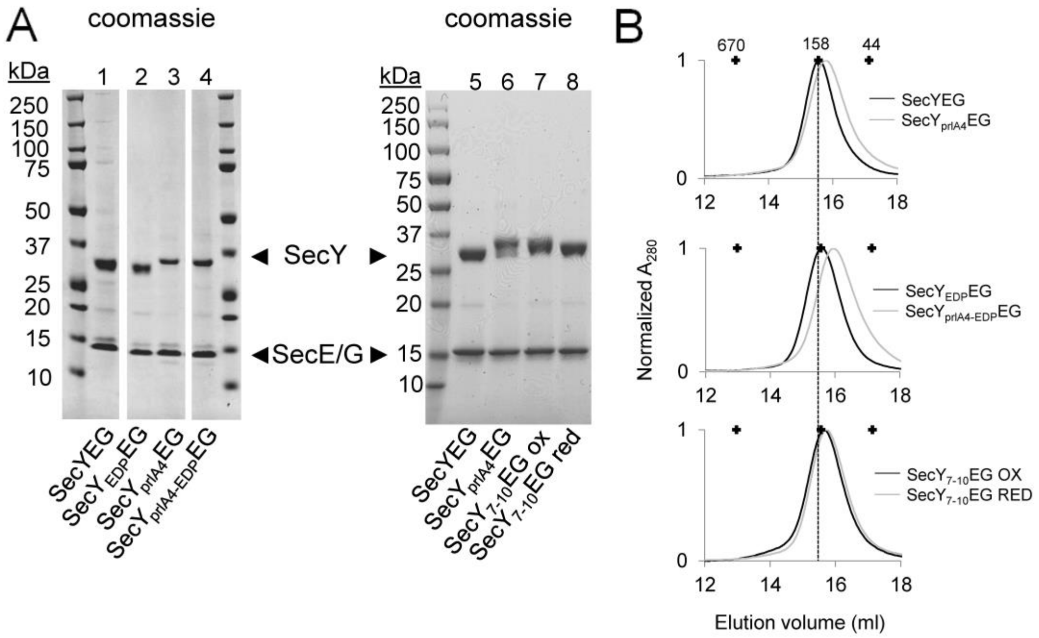

Figure S2. Related to Figure 2.

(A) SDS-PAGE analysis of the SecYEG variants stained with coomassie. The bands for SecY and SecE/G (which run at approximately the same position) and the MW markers are labelled on the side.

(B) Analytical size exclusion chromatography of the intact SecYEG complexes purified and run in detergent. Shown are the elution volumes of proteins of known molecular weight applied in order to align SecYEG complexes.

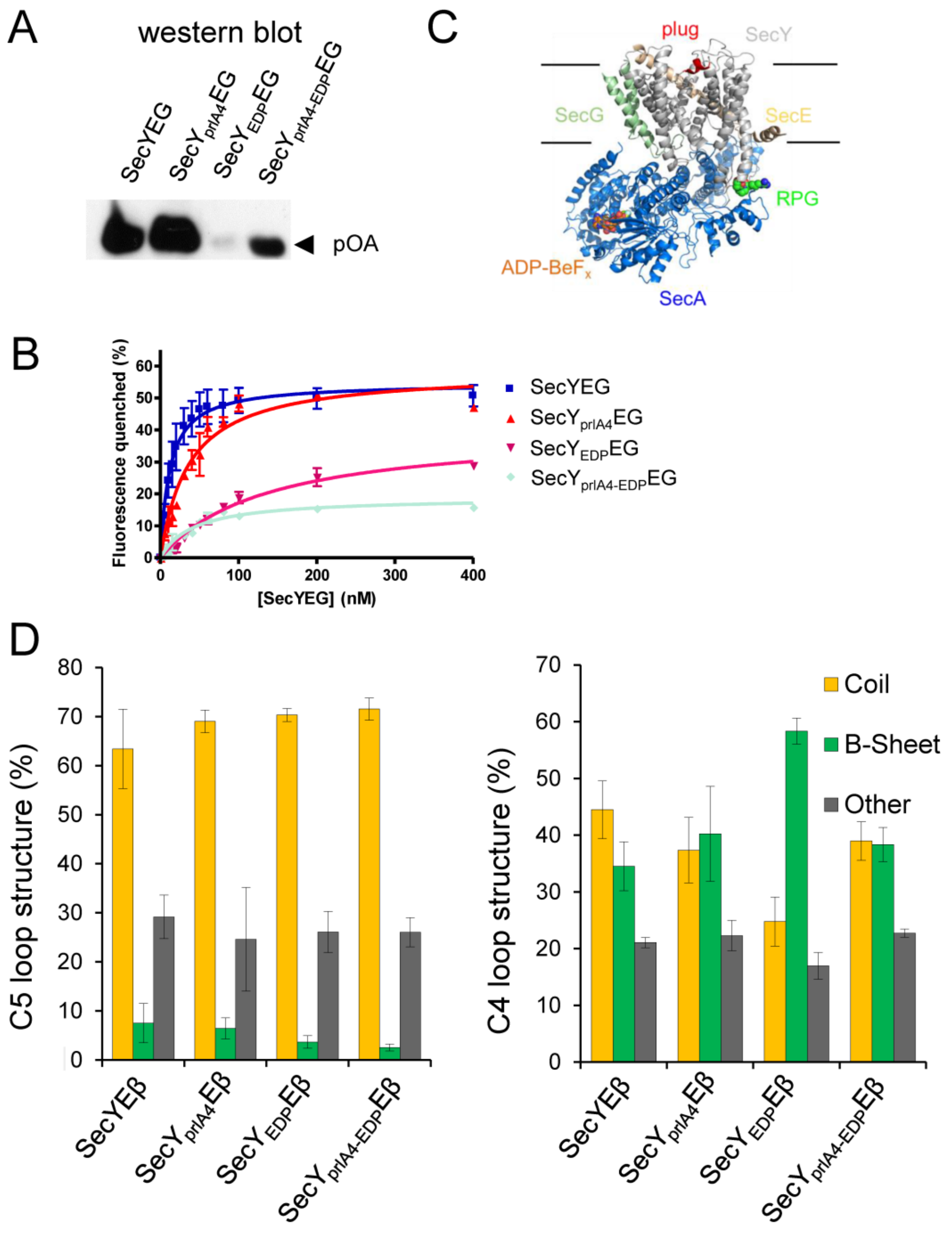

Figure S3. Related to Figure 2.

(A) Representative translocation assay for variants of SecYEG. Translocation efficiency is measured by western blotting with an antibody against a translocation substrate (pOA), and quantified as the percentage of the total pOA added to the assay. Three repeats of the assay were used for the quantification in Figure 2C.

(**B)** SecA binding assays using a fluorescently labelled SecA (SecA*). The fluorescence quench was plotted against different concentrations of SecYEG and the data was fitted to a tight binding equation. Error bars show the S.E.M of three repeats. The K_ds are plotted in Figure 2D.

(C) SecYEG-SecA (3DIN) (Zimmer et al., 2008), with the different subunits coloured and labelled accordingly. The RPG motif is shown as spheres, to illustrate its positioning with relation to SecA.

(D) MD simulations of SecYEβ mutants were analysed for secondary structure using the DSSP program (Joosten et al., 2011) on the SecY C5 loop (351-362 in *M. jannaschii* numbering) and C4 loop (residues 229-252 in *M. jannaschii* numbering). Yellow bars are unfolded regions (coil), green bars are β-sheets and grey bars are other types of secondary structure. Error bars show the S.E.M of three simulations.

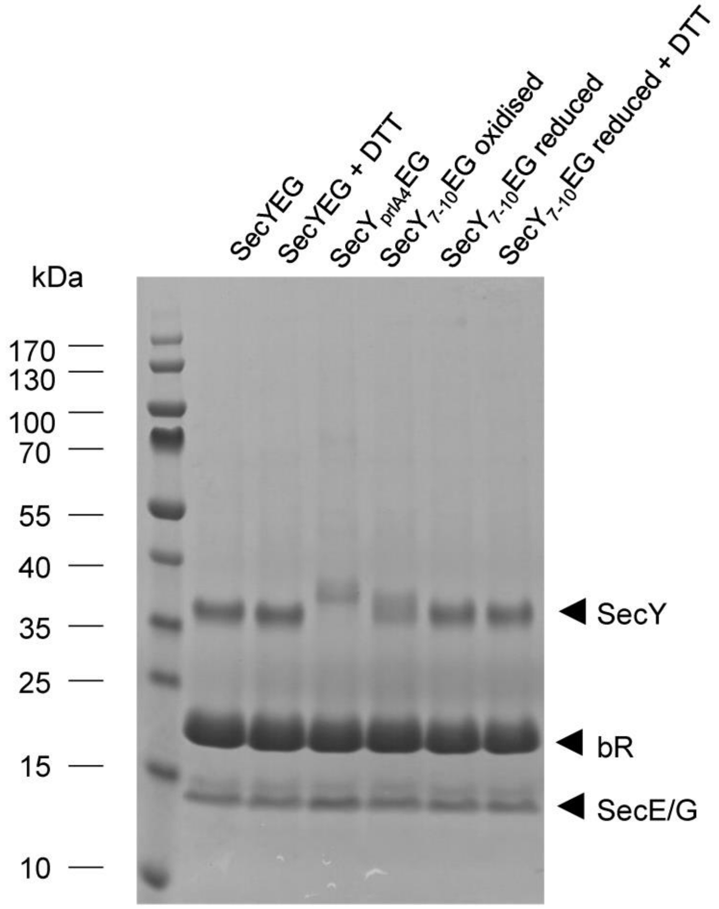

Figure S4. Related to Figure 3.

Analysis by SDS-PAGE and coomassie staining of proteo-liposomes (PL) reconstituted with SecYEG variants and bacteriorhodopsin (bR), with or without incubation with dithiothreitol (DTT). The MW markers, SecY, bR and SecE/G bands have been labelled on the side.

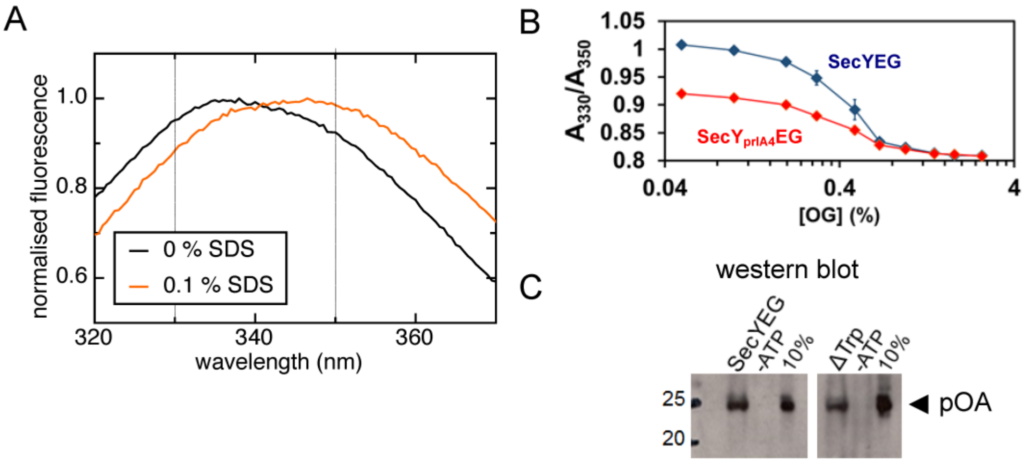

Figure S5. Related to Figure 4.

(A) Representative tryptophan fluorescence emission traces of SecYEG when excited at 288 nm, with either 0 % or 0.1 % SDS. Shown in vertical lines are the 330 and 350 nm positions used in secondary analysis. In general, SDS titration correlates with a redshift of the native SecYEG tryptophan residues, reflecting the exposure of these residues to solvent.

(B) The change in tryptophan fluorescence emission of standard SecYEG or SecY$_{prlA4}$EG in DDM, titrated with increasing concentrations of the partially destabilising detergent β-octyl glucoside. The y-axis shows the ratio of the emission amplitude at 330 and 350 nm. Note that as the x-axis is a log scale, so the 0 % β-octyl glucoside points cannot be shown. Error bars are S.E.M of three repeats.

(C) Translocation assay of PL reconstituted with SecYEG with all the native tryptophans replaced by phenylalanine. The western blot using an antibody against pOA visualises the successfully translocated substrate in the PL interior protected from protease k.

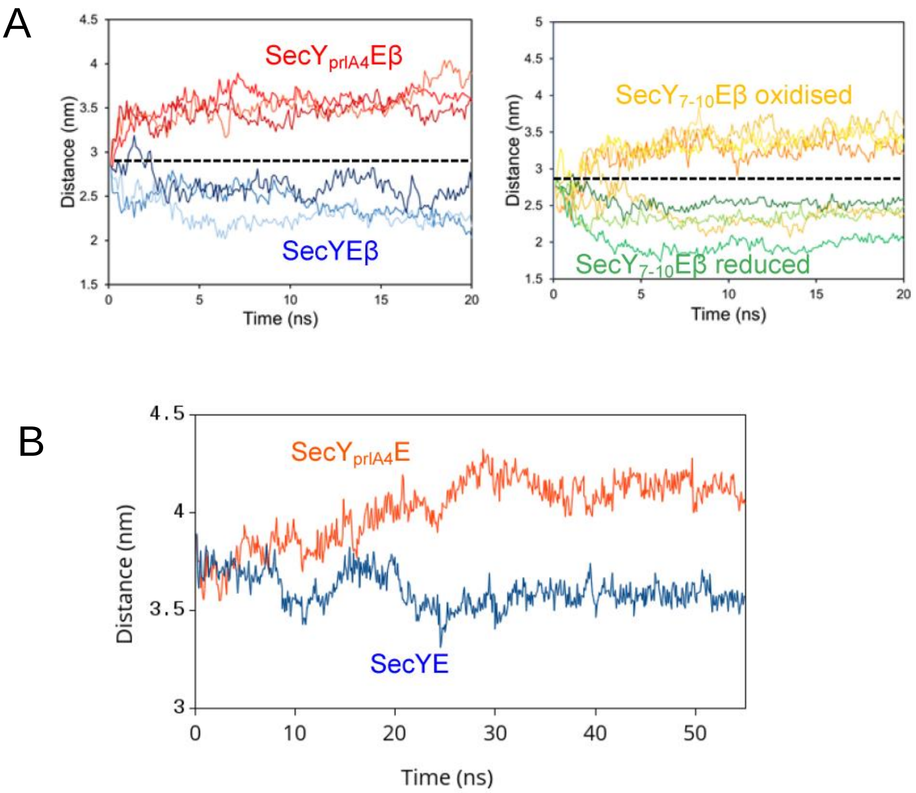

Figure S6. Related to Figure 5.

(A) Distance analysis between the SecE amphipathic helix and a rigid region of SecY, highlighted in Figure 5A; showing the first 20 ns of Figure 5B. Note that, with respect to the amphipathic helix of SecE, all of the simulations quickly diverge from the input model.

(B) Distance analysis between the SecE amphipathic helix and a rigid region of SecY (see Figure 5A) for MD simulations conducted on the gram negative *T. thermophilus* SecYE (2ZJS) (Tsukazaki et al., 2008). The standard SecYE, and a variant corresponding to the *E. coli* SecY$_{prlA4}$EG (*T. thermophilus* SecY$_{prlA4}$E) is in red. This analysis supports the data in Figure 5, and suggests that the observed shift of the SecE amphipathic helix towards the cytosol is conserved across both archaea (Figure 5) and gram-negative bacteria (shown here). Note that the region of SecE selected for distance analysis is the region containing W84, used for tryptophan fluorescence in Figure 4C.

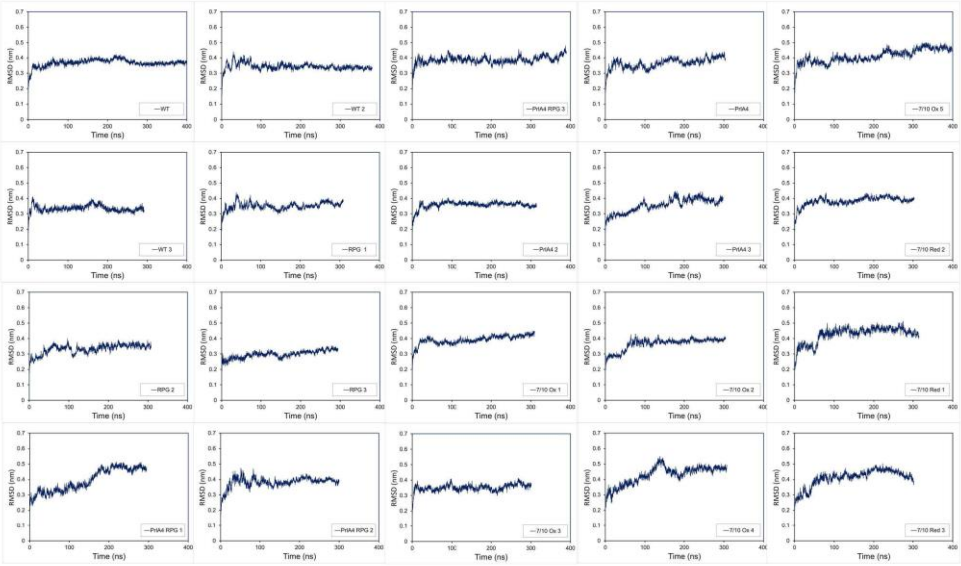

Figure S7. Related to Figure 5.

Root mean square deviation (RMSD) analysis for all of the *M. jannaschii* simulations. According to this analysis, the simulations are all stable after ~200 ns of simulation time.
