# Supplementary material for: Unlocking the Bacterial SecY Translocon
Source: Structure. 2016 Apr 5;24(4):518–27. doi: 10.1016/j.str.2016.02.001 (PMC4826270; doi:10.1016/j.str.2016.02.001)
Supplement: Document S2. Article plus Supplemental Information [file mmc2.pdf]

Article

Structure

Unlocking the Bacterial SecY Translocon

Graphical Abstract

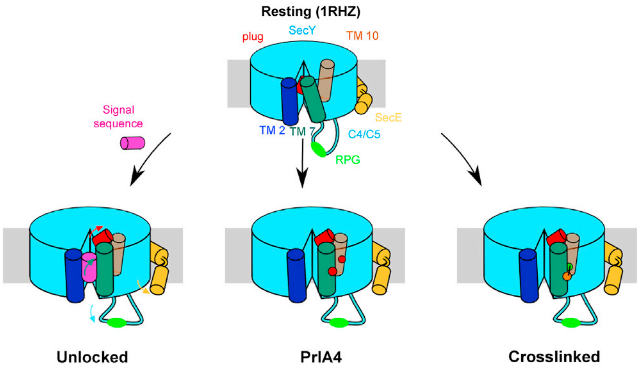

Authors

Robin A. Corey, William J. Allen, Joanna Komar, Simonas Masiulis, Sam Menzies, Alice Robson, Ian Collinson

Correspondence

ian.collinson@bristol.ac.uk

In Brief

Corey et al. have used biochemical and computational techniques to analyze conformational changes in the bacterial SecYEG translocon upon signal sequence binding. These structural effects are likely key to understanding how the translocon is primed for translocation by the concerted action of the signal sequence and the ATPase SecA.

Highlights

- Validation of previously observed signal sequence-induced "unlocking" of SecYEG
- Conformational changes upon SecYEG unlocking are relayed to SecA binding site
- Unlocking the translocon perturbs the interaction between SecY and SecE
- Conformational changes distinct between secretion and membrane protein insertion

Corey et al., 2016, Structure *24*, 518–527
April 5, 2016 ©2016 The Authors
http://dx.doi.org/10.1016/j.str.2016.02.001

Structure
Article

Unlocking the Bacterial SecY Translocon

Robin A. Corey,[1] William J. Allen,[1] Joanna Komar,[1] Simonas Masiulis,[1] Sam Menzies,[1] Alice Robson,[2] and Ian Collinson[1,*]
[1]School of Biochemistry, University of Bristol, University Walk, Bristol BS8 1TD, UK
[2]School of Biological and Chemical Sciences, Queen Mary University of London, Mile End Road, London E1 4NS, UK
*Correspondence: ian.collinson@bristol.ac.uk
http://dx.doi.org/10.1016/j.str.2016.02.001
This is an open access article under the CC BY license (http://creativecommons.org/licenses/by/4.0/).

SUMMARY

The Sec translocon performs protein secretion and membrane protein insertion at the plasma membrane of bacteria and archaea (SecYEG/β), and the endoplasmic reticular membrane of eukaryotes (Sec61). Despite numerous structures of the complex, the mechanism underlying translocation of pre-proteins, driven by the ATPase SecA in bacteria, remains unresolved. Here we present a series of biochemical and computational analyses exploring the consequences of signal sequence binding to SecYEG. The data demonstrate that a signal sequence-induced movement of transmembrane helix 7 unlocks the translocon and that this conformational change is communicated to the cytoplasmic faces of SecY and SecE, involved in SecA binding. Our findings progress the current understanding of the dynamic action of the translocon during the translocation initiation process. The results suggest that the converging effects of the signal sequence and SecA at the cytoplasmic face of SecYEG are decisive for the intercalation and translocation of pre-protein through the SecY channel.

INTRODUCTION

An essential part of the biogenesis of many proteins is the crossing or insertion into a lipid membrane, a process known as protein translocation. The bulk of translocation occurs at the ubiquitous and essential Sec translocon: Sec61αβγ in the eukaryotic ER membrane; SecYEG in the plasma membrane of bacteria, archaea (SecYEβ), and the thylakoid membrane of chloroplasts (Collinson et al., 2015). In bacteria, SecYEG associates with the cytoplasmic ATPase SecA (forming the translocase) to carry out post-translational translocation/secretion of proteins across the membrane (Brundage et al., 1990), in a process driven by ATP synthesis and enhanced by the transmembrane proton motive force (PMF). Conversely, membrane proteins are inserted into the bilayer upon direct interaction of SecYEG with the ribosome nascent chain complex (Beck et al., 2000). Auxiliary proteins including SecD, SecF, and YajC assist in the secretion process (Duong and Wickner, 1997), while YidC cooperates in the insertion of transmembrane helices (TMs) of membrane proteins (Scotti et al., 2000); together with the SecYEG core-translocon they form a holo-translocon complex (Samuelson et al., 2000; Schulze et al., 2014).

The resting state structure of the *Methanococcus jannaschii* translocon (SecYEβ) reveals a channel through the center of the complex, formed between the two halves of SecY: TMs 1–5 and 6–10 (Figure 1A, top left panel; van den Berg et al., 2004). In the resting state, this channel is blocked at the center by a ring of conserved hydrophobic residues and a short "plug" helix (Figure 1A, red helix). SecY is braced at the back by SecE, with one TM tilted across the membrane and an amphipathic helix flat along the cytoplasmic face of the membrane (Figure 1A, beige helices). For channel opening, SecE presumably relaxes and allows separation of the two halves of SecY along with the displacement of the plug (Tam et al., 2005; van den Berg et al., 2004; Flower et al., 1995). This opening creates a pathway for proteins through the middle of the channel for secretion, as well as sideways into the bilayer via a "lateral gate" (LG) for membrane protein insertion (van den Berg et al., 2004).

In bacteria, secretory proteins with a cleavable N-terminal signal sequence (SS) engage SecYEG and SecA at the plasma membrane, with the secretory protein still in an unfolded state (Arkowitz et al., 1993; Hartl et al., 1990). Once engaged, the SS is inserted into the membrane at the interface between the bilayer and the LG of SecY (Gold et al., 2013; Briggs et al., 1986; Mcknight et al., 1991; Plath et al., 1998); we have previously proposed that this interaction unlocks the translocon prior to channel opening through a series of conformational changes within SecY (Hizlan et al., 2012). Notably, TM 7 straightens by ~40° toward the center of the channel to contact TM 10 (Figure 1A, right panels, turquoise helix). This in turn displaces the plug (Figure 1A, right panels, red helix), and likely primes the channel for translocation.

Mutations of SecY have been identified that allow in vivo transport of pre-proteins with a defective SS (*prlA* mutants) (Smith et al., 2005; Emr et al., 1981; Derman et al., 1993). These mutants also exhibit increased translocation activity and are not further stimulated by the PMF (Nouwen et al., 1996). They possibly act by stabilizing the unlocked state of SecY, otherwise induced by SS binding; consistent with this, most *prlA* mutations map to TMs 7, 10, and the plug of SecY (van den Berg et al., 2004; Osborne and Silhavy, 1993). For example, the potent *prlA4* mutation ($SecY_{F286,/I408N}$ in *Escherichia coli*; Figure 1B, red circles) (Emr et al., 1981; Duong and Wickner, 1999) might promote the relocation of TM 7, thereby unlocking the complex in the absence of a functional SS.

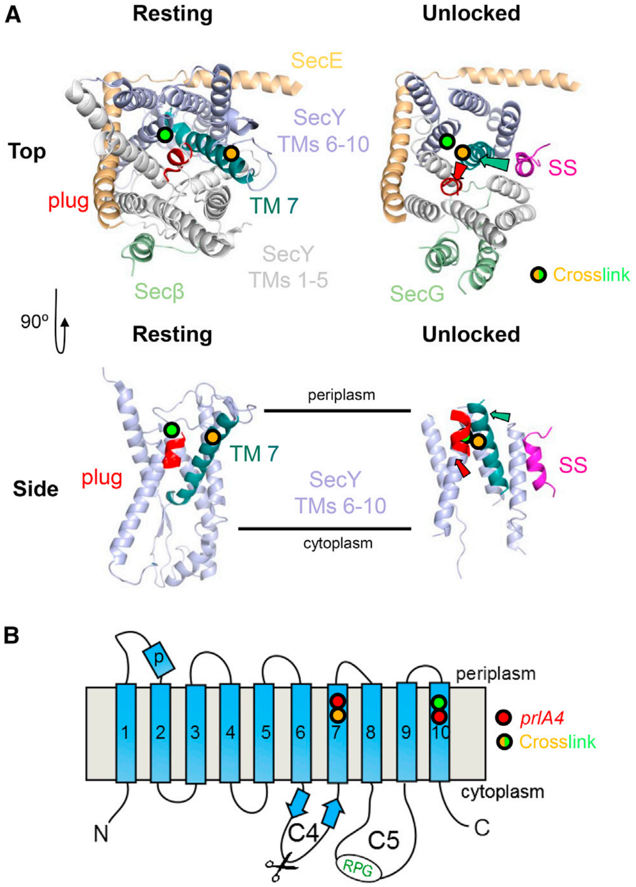

Figure 1. Structure of the SecY Complex and Previously Proposed Effects of Signal Sequence Binding

(A) Top left: *M. jannaschii* SecYEβ viewed from the outside (equivalent to the bacterial periplasm) (van den Berg et al., 2004). SecY TMs 1–5 are white; TMs 6–10 are blue; SecE is beige and Secβ is light green (equivalent to the *E. coli* SecG). In SecY, the plug (red) and TM 7 (turquoise) have been highlighted. Positions of SecY I284 and T404, which are crosslinked to form SecY$_{7\text{–}10}$EG, are shown as orange and green circles, respectively.

Top right: *E. coli* SecYEG bound to a signal sequence (SS; magenta) (Hizlan et al., 2012), coloring as in the equivalent view of the *M. jannaschii* SecYEβ (top left). The colored arrows highlight the substantial rearrangement of the plug and TM 7 upon SS binding.

Bottom left: as per the top left panel but viewed from the side and with TMs 1–5 of SecY, SecE, and SecG removed for clarity. The approximate position of the membrane is marked by black lines.

Bottom right: as per the bottom left panel but of the SS-bound structure, with the movement of the plug and TM 7 shown by colored arrows. The SS causes a distinctive tilting of TM 7.

(B) Schematic diagram of SecY showing the ten TMs, along with the plug domain (labeled "p") and loops C4 and C5. The position of the amino acid substitutions used in this study are shown as red circles for SecY$_{\text{prlA4}}$EG, orange and green circles for SecY$_{710}$EG, and green letters for the RPG motif. The trypsin cleavage site on SecY is indicated with a pair of scissors.

See also Figure S1.

A structure of *Thermotoga maritima* SecYEG bound and activated by SecA reveals further substantial changes in the SecY channel (Zimmer et al., 2008). Upon SecA binding, the two halves of SecY separate—characterized by a widening of the SecY LG—and TMs 8 and 9 move outward, producing a shift in the SecE amphipathic helix (Zimmer et al., 2008). SecA binds SecY at two cytoplasmic loops (C4 and C5; Figure 1B). The latter includes the highly conserved "RPG" site (SecY$_{\text{R357,P358.G359}}$ in *E. coli*), mutations of which perturb SecA interaction (Alami et al., 2007; Tam et al., 2005; de Keyzer et al., 2007) and thereby abolish translocation (Mori and Ito, 2001).

Table 1. Description of the SecYEG Variants Employed in the Study

SecYEG Variant	Substitutions
SecY$_{\text{prlA4}}$EG	SecY$_{\text{F286Y,I408N}}$
SecY$_{\text{EDP}}$EG	SecY$_{\text{R357E,P358D,G359P}}$
SecY$_{\text{prlA4-EDP}}$EG	SecY$_{\text{F286Y,I408N,R357E,P358D,G359P}}$
SecY$_{7\text{–}10}$EG	SecY$_{\text{I284C,T404C}}$

The name of the variant is shown in the left column and the corresponding point mutations in the right column.

It is not yet known how the SS and SecA-driven conformational changes prime SecYEG for transport, nor is it clear how they compare with those required for membrane protein insertion. Here we present a series of biochemical and biophysical analyses, supported by all-atom molecular dynamics (MD) simulations, aimed at investigating the mobility of key regions of the SecY protein channel during the initiation process. The data verify the physiological significance of the SS-induced displacement of TM 7, visualized in the absence of SecA (Hizlan et al., 2012), and provide further insights into the dynamic action of the SecY translocon during the early stages of protein secretion.

RESULTS

Production of Different States of SecYEG

A number of variants of *E. coli* SecYEG were produced (Table 1) to characterize the unlocking process: (1) the hyperactive SecY$_{\text{prlA4}}$EG variant (SecY$_{\text{F286Y/I408N}}$EG; red circles in Figure 1B), which allows translocation of substrates with a defective SS (Emr et al., 1981; Duong and Wickner, 1999); (2) the secretion-defective RPG variant, which perturbs SecA binding (SecY$_{\text{R357E/P358D/G359P}}$EG, hereafter referred to as SecY$_{\text{EDP}}$EG; "RPG" in Figure 1B) (Mori and Ito, 2001; Tam et al., 2005); and (3) a hitherto uncharacterized combination of the two (SecY$_{\text{prlA4-EDP}}$EG).

To trap the complex in the reported unlocked state (Hizlan et al., 2012), we engineered a unique cysteine pair into an otherwise cysteine-free mutant of SecYEG to crosslink TMs 7 and 10 of SecY (SecY$_{\text{I284C/T404C}}$EG, hereafter called SecY$_{7\text{–}10}$EG; Figures 1A and 1B, orange and green circles; Figure S1A, sticks). Membranes from *E. coli* cells expressing this construct were either reduced (1 mM tris(2-carboxyethyl)phosphine [TCEP]) or oxidized (1 mM Cu-phenanthroline) prior to purification of SecYEG in detergent. The concentration of free thiols in the purified protein demonstrates that the oxidized form is >85% crosslinked in detergent solution (Figure S1B).

The anomalous behavior of SecY during SDS-PAGE is shared by many other membrane proteins and probably arises due to its partially folded state in SDS and, hence, faster migration through the gel and significantly reduced apparent molecular weight

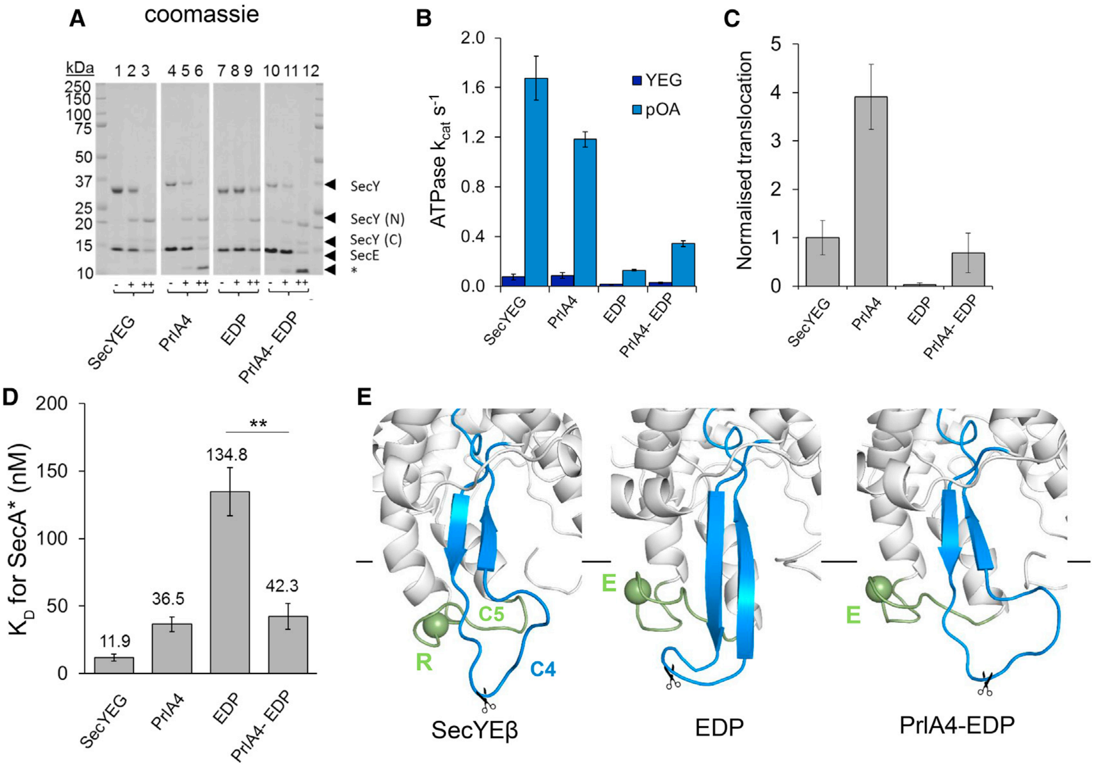

Figure 2. Competing Effects of the Activating Variant *prlA4* and Inactivating Variant SecY$_{EDP}$EG

(A) SecYEG, SecY$_{prlA4}$EG (PrlA4), SecY$_{EDP}$EG (EDP), and SecY$_{prlA4\text{-}EDP}$EG (PrlA4-EDP) were subjected to trypsin digestion followed by Coomassie-stained SDS-PAGE to examine the effect of different amino acid substitutions on the conformation of the C4 loop of SecY (the site of a prominent tryptic cleavage site). Samples are either untreated (−) or treated with 0.075 μg/ml (+) or 0.75 μl/ml (++) trypsin. The major bands correspond to full-length SecY, the N-terminal cleavage fragment, the C-terminal fragment, SecE, and a lower band for both SecE breakdown products and secondary SecY cleavage products (asterisk).

(B) The rate of ATP turnover (k_{cat}) is shown for SecA bound to different variants of SecYEG reconstituted into PL (purple), and following addition of a translocation substrate pOA (blue). ATP turnover is diminished by the C5 substitutions (SecY$_{EDP}$EG), but is then restored when combined with the *prlA4* mutations. Error bars denote SEM of three repeats.

(C) Translocation of pOA into PL reconstituted with different SecYEG variants. Translocation efficiencies were determined by western blotting against pOA following a protease K protection assay (transported pOA is in the PL interior and protected from proteolysis); for a representative blot see Figure S3A. Results were quantified against a non-protease K-treated control and normalized to standard SecYEG, shown with SEM of three repeats. As in (B), the abolished activity of SecY$_{EDP}$EG (EDP) is rescued by the *prlA4* mutations.

(D) Affinities of SecYEG for SecA in detergent solution, determined through quenching of a fluorescent marker on SecA (SecA*). The decrease in fluorescence was plotted and fitted to tight binding equations (raw data are shown in Figure S3B). The calculated K_D values are shown with SEM of three runs. **p = 0.004.

(E) Representative structures of the cytoplasmic face of SecY (cartoon representation) following MD simulations of SecYEG, SecY$_{EDP}$EG, and SecY$_{prlA4\text{-}EDP}$EG. Structures are viewed from the side, with the cytosolic face indicated by the black line. The C4 loops are shown in light blue, the C5 loops green, and the rest of SecY white. In each panel, the position of the Cα of the conserved arginine in the RPG motif (mutated to glutamic acid in the middle and right-hand panels) is shown as a green sphere and labeled "R" or "E" accordingly. The approximate position of the trypsin cleavage sites is indicated by a pair of scissors. These structural changes are quantified in Figure S3D.

(MW) (~30 kDa; Figure S2A) compared with the actual MW (~48 kDa). Interestingly, SecY$_{prlA4}$ has a slight, but consistent, increase in apparent MW (~34 kDa; Figure S2A); this presumably correlates with a less compact, or more flexible, state. Note that oxidized SecY$_{7\text{–}10}$ also has reduced mobility, albeit less pronounced (Figure S2A; ~33 kDa), suggesting that the loosening observed in SecY$_{prlA4}$ can be partially achieved by displacing TM 7 with a crosslink to TM 10.

Similar effects were observed on the whole SecYEG complex following analysis by size-exclusion chromatography (Figure S2B). Evidently, the loosening of SecYEG brought about by SecY$_{prlA4}$, and to a lesser extent SecY$_{7\text{–}10}$ (and hence the movement of TM 7), may be correlated with the unlocking and activation process.

The Inactivation Caused by C5 Loop Variants Are Reversed by Substitutions inside the SecY Channel

Exposure of SecYEG to trypsin results in proteolysis at the cytoplasmic face of SecY, with cleavage occurring predominantly on the C4 loop (Brundage et al., 1990), also known to be important for the interaction of SecY with SecA and the ribosome (Figure 1B, scissors). Using low and high concentrations of trypsin (Figure 2A + and ++), standard SecY is either partially or completely cleaved, respectively (Figure 2A, lanes 1–3). Trypsin sensitivity is largely unaffected by the SecY$_{prlA4}$EG substitutions (Figure 2A, PrlA4 in lanes 4–6), but considerably reduced with the SecY$_{EDP}$EG substitutions, which are on the adjacent loop (C5) (Figure 2A, EDP in lanes 7–9). The reduced trypsin sensitivity suggests that SecY$_{EDP}$EG brings about a

broad conformational change affecting both loops C5 (containing the RPG motif) and C4 (containing the tryptic site). This effect is reversed when $SecY_{EDP}EG$ is combined with the *prlA4* mutations ($SecY_{prlA4\text{-}EDP}EG$) (Figure 2A, PrlA4-EDP in lanes 10–12), indicating that the perturbation of TM 7 (by *prlA4*) restores the native conformation of the cytosolic regions of SecYEG. Thus, TM 7 is conformationally coupled to the surface of SecYEG.

Activation of SecYEG through Movement of TM 7 toward TM 10 by *prlA4* Overrides the Loss of Function Caused by the RPG Variant

Proteoliposomes (PL) containing SecYEG, $SecY_{prlA4}EG$, $SecY_{EDP}EG$, and $SecY_{prlA4\text{-}EDP}EG$ were used to reconstitute and monitor ATP-driven protein translocation. The results show that, as expected (Mori and Ito, 2001), $SecY_{EDP}EG$ displays a dramatic loss in both SecA activation and translocation activity (Figures 2B, 2C and S3A). Interestingly, as with trypsin sensitivity, this effect is partially ameliorated by the *prlA4* mutations (Figures 2B and 2C, $SecY_{prlA4\text{-}EDP}EG$). Thus, the effects of *prlA4* are dominant to the inactivating $SecY_{EDP}EG$ with respect to transport activity.

Next, the affinity of SecYEG for SecA was measured using a fluorescent probe on SecA (hereafter SecA*) which, when bound by a non-hydrolyzable analog of ATP (AMP-PNP), is quenched upon SecY binding (Deville et al., 2011) (Figure S3B). SecA* binds tightly to standard SecYEG (K_D ~12 nM; Figures 2D and S3B), with the affinity slightly lower for $SecY_{prlA4}EG$ (K_D ~36 nM; Figure 2D). This is perhaps surprising, as previous results have indicated a higher affinity for $SecY_{prlA4}EG$ (de Keyzer et al., 2002; van der Wolk et al., 1998). A possible explanation for this disparity is the use in the previous study of crude inner membrane vesicles (IMVs) rather than purified components; experiments conducted in IMVs may be influenced by auxiliary translocon components (e.g., SecDF) or unknown effector proteins.

The affinity of SecA for $SecY_{EDP}EG$ is more than 10-fold weaker than for standard SecYEG (K_D ~135 nM; Figure 2D); this is unsurprising, as the RPG site is located on the SecY-SecA interface (Figure S3C). Once again, however, this effect is reversed when the $SecY_{EDP}EG$ variant is paired with the *prlA* mutations (K_D ~42 nM; Figure 2D).

The RPG Substitutions Perturb the Adjacent C4 Loop

To investigate the structural changes associated with the $SecY_{EDP}EG$ substitutions, we ran all-atom MD simulations using the *M. jannaschii* SecYEβ crystal structure as starting coordinates (PDB: 1RHZ; van den Berg et al., 2004). Three simulations each of SecYEβ and the corresponding variants $SecY_{prlA4}E\beta$, $SecY_{EDP}E\beta$, and $SecY_{prlA4\text{-}EDP}E\beta$ were run for 300–400 ns to provide suitable sampling.

It is apparent from the MD data that the $SecY_{EDP}E\beta$ substitutions on loop C5 have little effect on the conformation of this loop (Figures 2E and S3D). Interestingly, they do produce considerable secondary structural change on the adjacent C4 loop—which contains the primary trypsin cleavage site—with a marked increase in β-sheet composition (Figures 2E and S3D, right panel). $SecY_{prlA4}E\beta$ and $SecY_{prlA4\text{-}EDP}E\beta$ are largely unchanged when compared with the native SecYEβ. Therefore, the MD results are consistent with the trypsin sensitivity data (Figure 2A), showing that the $SecY_{EDP}E\beta$ substitutions on the C5 loop are transmitted to the adjacent C4 loop, and that these effects are reversed by the distant *prlA4* mutations.

The activating *prlA4* mutations are consistently dominant over the inactivating $SecY_{EDP}EG$, demonstrating that the impaired function of $SecY_{EDP}EG$ is not simply a loss of a direct contact site for SecA. The data here reveal a functional complementation between regions deep within the protein channel (*prlA4* sites; TM 7 and TM 10) and the cytosolic surface, typical of linked conformational changes over long range.

Straightening of TM 7 toward TM 10 Activates SecYEG

The $SecY_{7\text{–}10}EG$ variant (Figures 1 and S1A) comprises an engineered cysteine pair between TMs 7 and 10 with the two native cysteines in SecY replaced with serine (which has no effect on the function of SecYEG; Kaufmann et al., 1999). This strategically placed thiol pair was designed to trap the translocon in the unlocked state, as seen with SS binding (Hizlan et al., 2012), and is thought also to be favored by $SecY_{prlA4}EG$.

$SecY_{prlA4}EG$ has an enhanced translocation activity but is unaffected by the PMF (Nouwen et al., 1996), so $SecY_{7\text{–}10}EG$ should exhibit similar properties. To this end, we carried out translocation assays on each variant in PL with and without PMF stimulation, achieved by co-reconstituting SecYEG with the light-driven proton pump bacteriorhodopsin (bR; Figure S4), as described previously (Schulze et al., 2014). As expected, $SecY_{prlA4}EG$ is activated with respect to translocation compared with the standard form of SecYEG (Figure 3A, "−Light" lanes), and this activity is not further enhanced by the PMF (Figure 3A, "+Light" lanes). Unexpectedly, the cysteine pair of $SecY_{7\text{–}10}EG$ alone has the same effect on translocation activity as $SecY_{prlA4}EG$, whether oxidized or reduced. However, when TM 7 is permanently fixed to TM 10 by a disulfide bond the channel is found in the activated state (as it is with the $SecY_{prlA4}EG$ and $SecY_{7\text{–}10}EG$ reduced). The fact that the disulfide crosslink retains this activated state suggests that the proximity of TMs 7 and 10 is a feature of this activation process (although it can also be achieved without the permanent crosslink). Therefore, the movement of TM 7 toward TM 10 activates the SecYEG complex, presumably in the manner proposed for $SecY_{prlA4}EG$, i.e., through the displacement of TM 7.

The Activation Pathways for Protein Secretion and Membrane Insertion Are Distinct

Experiments were conducted to establish whether the activation and unlocking process described for protein secretion also holds true for membrane protein insertion. The perturbation of TM 7 in $SecY_{prlA4}EG$ or $SecY_{7\text{–}10}EG$ did not result in increased insertion activity of a model substrate, F_O(a) (Figure 3B). Therefore, for this substrate the activation pathways for membrane protein insertion are distinct to those seen for protein secretion, at least with respect to the mobility of TM 7.

The Movement within SecY of TM 7 toward TM 10 Is Relayed to the Amphipathic Helix of SecE

SecYEG activation has previously been linked to destabilization of the complex (Duong and Wickner, 1999), potentially by promoting a separation of the two halves of SecY. We reasoned that this effect could also be induced by low levels of a

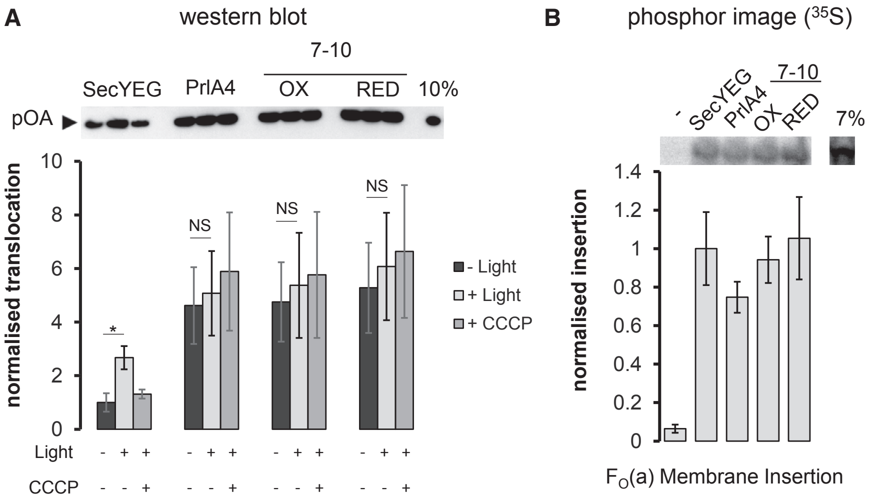

Figure 3. Comparative Secretion and Membrane Protein Insertion Activity of the SecYEG Variants

(A) Post-translational translocation of the pre-protein substrate pOA into the interior of PL co-reconstituted with SecYEG and the light-driven proton pump bacteriorhodopsin (BR), with or without PMF (generated by bR: + Light or – Light). The transport efficiencies were determined by western blotting against pOA, and normalized to SecYEG without light; the SEM is shown for three repeats. *p = 0.018; NS, not significant.

(B) Co-translational membrane protein insertion of subunit a of the F_1F_O-ATP synthase (F_O(a)) into the membranes of PL containing variants of SecYEG. The insertion levels were determined by the quantification of radiolabeled [^{35}S]methionine incorporated into the newly synthesized and membrane inserted F_O(a) substrate, averaged over three runs with SEM shown by the error bars.

denaturant, such as SDS. To monitor the effect of denaturation, we exploited the sensitivity of intrinsic tryptophan fluorescence to conformational changes within proteins: increasing concentrations of SDS were titrated into SecYEG, and tryptophan emission spectra measured.

The general effect of SDS addition is a red shift in emission, probably due to increasing solvent exposure of tryptophan residues as the complex unfolds (Figure S5A). This shift can be expressed as a ratio of the amplitude of the emission at 330 nm and 350 nm (A_{330}/A_{350}) and plotted against increasing concentrations of SDS (Figure 4A). The profiles for standard SecYEG and the reduced $SecY_{7–10}EG$ (Figure 4A, blue and green traces, respectively) reveal a distinct transition at ~0.01% SDS. The unlocked forms of the complex, i.e., $SecY_{prlA4}EG$ and oxidized $SecY_{7–10}EG$ (Figure 4A, red and orange traces, respectively), exhibit a significant reduction in A_{330}/A_{350} without SDS, and a diminished transition at ~0.01% SDS. This effect is also observed when substituting SDS for a β-octyl glucoside (Figure S5B), for which SecYEG destabilization and dissociation has previously been characterized (Duong and Wickner, 1999). Therefore, the data indicate that the activated complexes are already in a partly destabilized form.

E. coli SecYEG has eight native tryptophan residues (four in SecY, three in SecE, and one in SecG; see Figure 4B). To localize the conformational change seen in Figure 4A, we created variants in which only one native tryptophan was retained, with the others substituted with phenylalanine, the product of which was still fully functional (Figure S5C). Each variant was individually examined and the observed transition was located to W84 (Figure 4C, blue line), positioned on the amphipathic helix of SecE (Figure 4B, W84). In a *prlA4* background no transition is apparent: W84 already appears to be exposed to solvent even in the absence of denaturant (Figure 4C, pink line). When W84 was substituted with phenylalanine but the other native tryptophan residues retained, as expected the amplitude of the denaturation curve was significantly diminished (Figure 4D, black line). This effect mirrors $SecY_{prlA4}EG$ retaining all eight native tryptophan residues, wherein W84 is unresponsive to denaturant. While the remaining seven tryptophan residues play a small role in this transition, the W84 effect is clearly dominant.

It is evident from the data that the decreased transition of $SecY_{prlA4}EG$, caused by the ready exposure of W84, is also apparent when TM 7 is crosslinked to TM 10 (Figure 4A). Therefore, the movement of TM 7 to TM 10 brings about a conformational change involving the amphipathic helix of SecE (containing W84), which is also apparent upon complex destabilization.

Mobility of the Amphipathic Helix of SecE in Response to the Perturbation of TM 7

To help understand the conformational effects arising from TM 7 perturbation, we ran MD simulations, as above, using *M. jannaschii* SecYEβ (PDB: 1RHZ; van den Berg et al., 2004), and the corresponding variants $SecY_{prlA4}E\beta$, oxidized $SecY_{7–10}E\beta$, and reduced $SecY_{7–10}E\beta$.

Post-simulation structural alignment of the different complexes reveals that the region with highest variability is the amphipathic helix of SecE (Figure 5A), near the position of $SecYE_{W84}G$ (Figure 5A, cyan asterisk). The helices in the unchanged SecYEβ simulations display an upward tilt (i.e., toward the periplasm) compared with the input structure, whereas the $SecY_{prlA4}E\beta$ simulations display a downward tilt toward the cytoplasm (Figure 5A, blue and red, respectively). When TM 7 is fixed by crosslinking to TM 10, the position of the amphipathic helix resembles that of $SecY_{prlA4}E\beta$ (Figure 5A, orange), whereas with no crosslink the helix location resembles the standard SecYEβ form (Figure 5A, green). Note that the SecYEβ and $SecY_{prlA4}E\beta$ simulations in Figure 5A are the same as those presented in Figure 2E.

Distance analysis between the N-terminal tip of the SecE amphipathic helix and a rigid region of SecY (Figure 5A, yellow box) confirms that the effect is consistent across all three repeats, or in four of five repeats in the case of oxidized $SecY_{7–10}EG$ (Figure 5B), and is apparent even in the early (20 ns) stages of the simulations (Figure S6A). Of the five repeats for the oxidized $SecY_{7–10}EG$ trajectories (with the relocated TM7), one behaves like the reduced states (marked with a light orange arrow); thus, the analysis predicts that the position of the amphipathic helix of SecE has a strong, but not absolute,

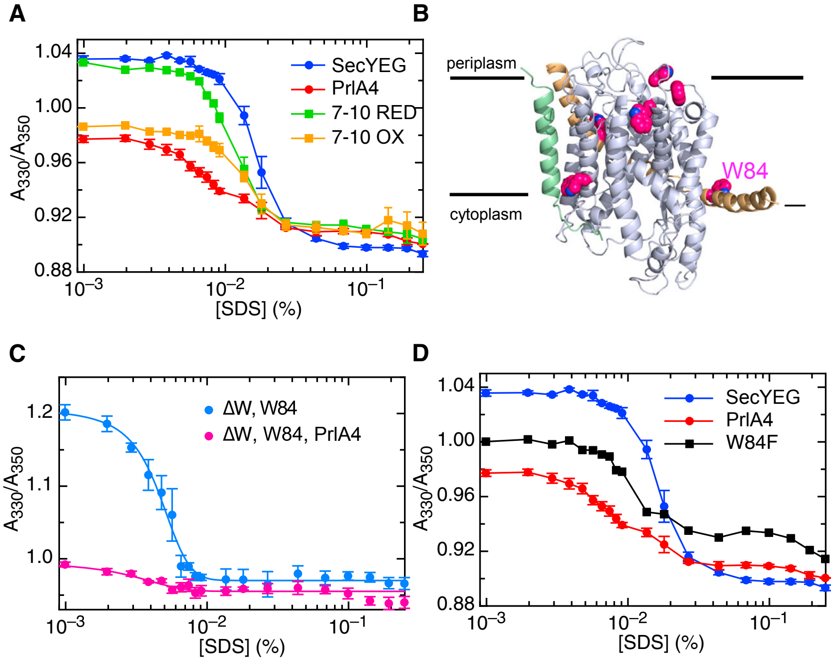

Figure 4. Conformational Change of SecYEG Monitored by the Intrinsic Fluorescence of the Native Tryptophan Residues

(A) Changes in tryptophan fluorescence emission were recorded upon SDS titration for different SecYEG variants in detergent solution. The y axis shows the ratio of the fluorescence emission at 330 and 350 nm after excitation at 288 nm. Error bars are the SEM of three repeats.

(B) Positions of native tryptophan residues in SecYEG (carbon atoms as magenta spheres, nitrogen atoms as blue spheres), shown on an *E. coli* homology model based on the *M. jannaschii* SecYEβ structure (1RHZ; van den Berg et al., 2004). Note that one of the *E. coli* tryptophan residues on SecE is missing as this TM is not present in *M. jannaschii*. The position of W84 is marked.

(C) Fluorescence unfolding profile of SecYEG as in (A), but with the single tryptophan variant ($SecY_{W84}EG$; W84) and all other tryptophans changed to phenylalanine, in either standard SecYEG or *prlA4* background. Error bars are the SEM of three repeats.

(D) Fluorescence unfolding profile of SecYEG as in (A), but with W84 substituted with phenylalanine and all other tryptophan residues retained (black line). The data for the experiment with standard SecYEG and $SecY_{prlA4}EG$ are shown for comparison (the same data as in A). Error bars are the SEM of three repeats.

dependence on the location of TM 7. To ensure this was not an archaea-specific artifact, we also ran simulations using SecYE from the Gram-negative *Thermus thermophilus* (PDB: 2ZJS; Tsukazaki et al., 2008). For simulations of standard SecYE and a $SecY_{prlA4}E$ equivalent, the SecE amphipathic helix behaves in a remarkably similar way to the corresponding feature of *M. jannaschii* (Figure S6B). Indeed, the SecE region most perturbed in these simulations is the region containing the $SecYE_{W84}G$ equivalent.

Note that, with respect to the SecE amphipathic helix, the MD simulations all diverge from the input structure (Figure 5B, black dashed line), despite having generally low root-mean-square deviations (Figure S7). This is possibly due to the presence of lipid bilayer in the MD simulations, absent in the crystal structure.

The simulation data are consistent with the tryptophan fluorescence analysis that localizes the primary effect of TM 7 movement to W84—on the amphipathic helix of SecE. Together, the results suggest that the change in conformation of TM 7 at the center of the channel, normally induced by SS binding, is coupled to the cytosolic surface of SecE. These SecE conformational changes differ from those reported in the SecA-bound SecYEG crystal structure (PDB: 3DIN, Figure 5C; Zimmer et al., 2008), suggesting a distinct role for the SecE amphipathic helix during SS-induced unlocking and SecA-mediated activation of the translocon.

DISCUSSION

This study explores the dynamic action of SecYEG during the initial stages of protein translocation, and builds on previous structural data of the *E. coli* SecYEG complex bound by a preprotein mimic (Hizlan et al., 2012). In this previous study, we proposed that the association of the SS with SecYEG causes a conformational change involving TM 7, TM 10, and the plug of SecY, which we likened to an "unlocking" event. Here, we trap the translocon in this unlocked state with use of an engineered disulfide crosslink between TMs 7 and 10 of SecY ($SecY_{7–10}EG$). Through biochemical and computational characterization of both this $SecY_{7–10}EG$ variant and the hyperactive $SecY_{prlA4}EG$, we demonstrate that the movement of TM 7 toward TM 10 unlocks the channel and increases the translocation activity of SecYEG. As the activating effect of the *prlA4* mutations likely arises from partial destabilization of the SecYEG complex (Duong and Wickner, 1999), this TM 7 configuration could be acting by a general dislocation of SecY, readying it for channel opening and protein translocation.

Curiously, when SecYEG is in a membrane environment, the engineered cysteines in TMs 7 and 10 alone (uncrosslinked) stimulate protein translocation, similar to $SecY_{prlA4}EG$ and the crosslinked variant $SecY_{7–10}EG$ (Figure 3A). However, when in a non-membrane environment they fail to replicate the unlocked state characteristic of the activated $SecY_{prlA4}EG$ (Figure 4A). Most likely, TM 7 is delicately poised between the two states, and for the uncrosslinked variant the known activating properties of the membrane bilayer (Gold et al., 2010; Lill et al., 1990; Robson et al., 2009) are sufficient to tip the balance toward the unlocked state, whereas in the absence of a biological membrane (i.e., in detergent solution) the crosslink is required for activation. This is not without precedent, as clear conformational differences have been observed in the transmembrane region of SecYEG in structures of the complex determined in detergent compared with the native lipid environments (Bostina et al., 2005).

Our analyses indicate that the unlocking mechanism, involving TM 7 at the center of SecY, is transmitted to the cytosolic face of

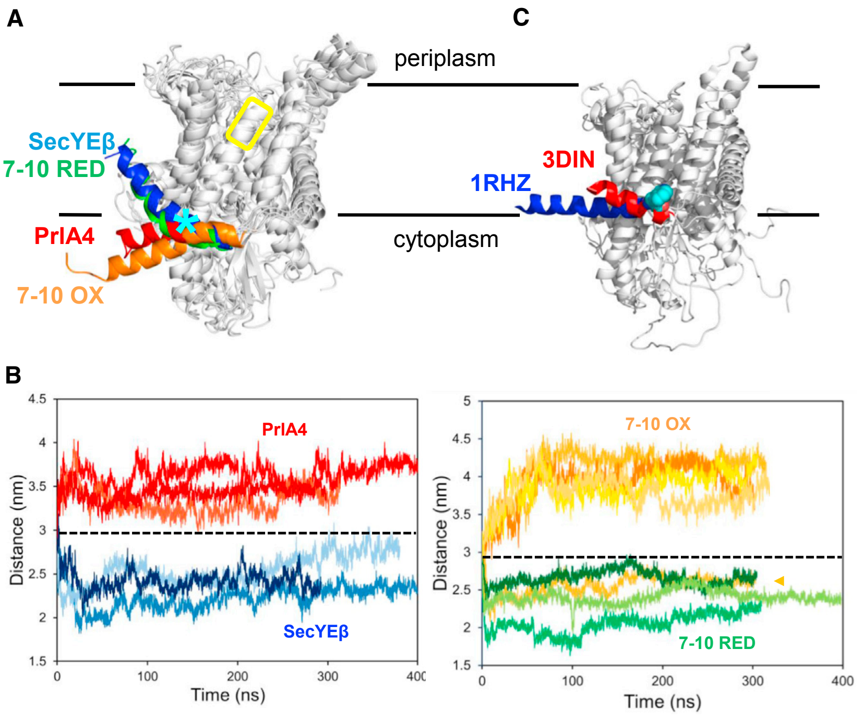

Figure 5. Molecular Dynamics Simulations of the SecY Complex

(A) Post-simulation snapshots of the *M. jannaschii* SecYEβ variants with the whole complex shown in gray except the amphipathic helix of SecE, which is colored according to variant: standard SecYEβ in blue; PrlA4 in red; oxidized SecY$_{7\text{–}10}$Eβ in orange and reduced SecY$_{7\text{–}10}$Eβ in green. The immobile region of SecY used for distance analysis in (B) is highlighted with a yellow box. The equivalent position of W84 is marked with a cyan asterisk.

(B) Distance analysis between the SecE amphipathic helix and a rigid region of SecY (yellow box in A). The first 20 ns of simulation are shown in detail in Figure S6A. The distance for the input structure is shown by the black dotted line. In the right panel, the single oxidized SecY$_{7\text{-}10}$EG simulation, which resembles the reduced states, is marked with a light orange arrow.

(C) Crystal structures of the SecY complex alone, *M. jannaschii* SecYEβ (1RHZ) (van den Berg et al., 2004), and SecY bound to SecA, *T. maritima* SecYEG-A (3DIN) (Zimmer et al., 2008) (SecA not shown). The amphipathic helix of SecE is shown in both structures, respectively in blue and red, and the equivalent key tryptophan residue of the *E. coli* SecY-W84 (W20 in *T. maritima*) is highlighted by cyan spheres.

SecYEG, with the most prominent structural change in the SecE amphipathic helix (Figures 4 and 5), which is well conserved between archaea and bacteria (Enno et al., 1994). In addition, through analysis of SecY$_{EDP}$EG with and without *prlA4*, it is clear that channel unlocking leads to a restructuring of the cytoplasmic loops of SecY (Figure 2). These loops are universally important in the interaction of SecY with translocation partners, such as the ribosome or SecA, with the R357 position completely conserved across all three domains of life. It is conceivable that the region containing the RPG motif acts as a conserved coupling switch for the activation of the translocon, either by SecA or the ribosome, or even analogous translocation partners in organisms lacking SecA. Indeed, the functional complementation of bacterial SecY by the archaeal counterpart (Auer et al., 1991) supports the existence of a common mechanism for activation.

The reported conformational coupling between SS and SecA binding is consistent with the observed transactivation of SecA by the SS (Gouridis et al., 2009), which occurs via SecYEG (Hizlan et al., 2012). Indeed, structural studies have shown that both SS and SecA binding have major effects on SecYEG (Hizlan et al., 2012; Zimmer et al., 2008): in the LG, plug, and C4 and C5 loops of SecY, and the amphipathic helix of SecE (Collinson et al., 2015). The perturbation of the amphipathic helix of SecE shown here, induced by the SS via TM7, might release the SecE brace around SecY and promote channel opening, as originally proposed on the basis of the SecYEβ structure (van den Berg et al., 2004). This concerted action of SS and SecA presumably primes SecYEG for the introduction of pre-protein, and acts as a prelude to ATP- and PMF-driven translocation (Figure 6).

The events described here relate to SecA-dependent bacterial secretion. Post-translational secretion through the eukaryotic counterpart (Sec61), however, is driven by a different mechanism involving the "pulling" action of an Hsp70 homolog (BiP) from within the ER lumen (Brodsky et al., 1995). Nevertheless, the unlocking mechanism shown here may be preserved, as both TM 7 and the location of the SS binding site are highly conserved (Plath et al., 1998; van den Berg et al., 2004). In eukaryotes, the luminal loop between TMs 7 and 8 is substantially longer than the bacterial equivalent in the periplasm and is known to interact with BiP (Schauble et al., 2012). It is therefore plausible that protein secretion in the eukaryotic counterpart could also be initiated by the perturbation of TM 7, brought about by the concerted action of SS, the BiP ATPase, and/or the ribosome. The recent structure of the mammalian Sec61 complex associated with the ribosome and nascent secretory protein (Voorhees and Hegde, 2016) is most likely captured beyond the initiation phase, showing the channel in the open state with widened LG and shifted TM 7, equivalent to that shown in Figure 6 (open).

EXPERIMENTAL PROCEDURES

Chromatography media were purchased from GE Healthcare. Detergents were obtained from Glycon and lipids from Avanti Polar Lipids. NuPAGE gels were bought from Life Technologies. Unless stated, all other materials were supplied by Sigma-Aldrich.

Protein Production

Point mutations were introduced using the QuikChange protocol (Stratagene) and confirmed by sequencing. The SecYEG variants were expressed and purified by procedures developed for the standard complex (Collinson et al., 2001). In brief, SecYEG-pBAD was expressed in C43 *E. coli* cells, which were lysed and the membrane fraction isolated by centrifugation; the SecYEG protein was solubilized from the membrane fraction using 1% DDM (*n*-dodecyl β-D-maltoside) and then purified using nickel chromatography

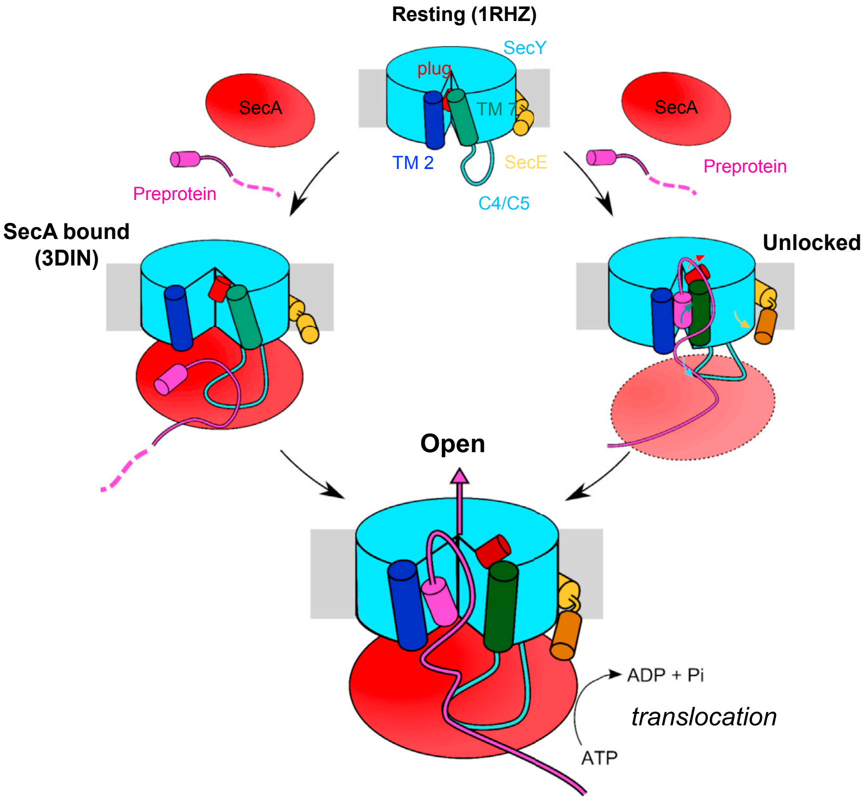

Figure 6. Model of SecYEG Unlocking and Activation by the Cooperative Action of the Signal Sequence and SecA

Resting: based on the closed structure, as seen in 1RHZ (van den Berg et al., 2004), with SecY in light blue, the lateral gate (LG) helices TM 2 and TM 7 in dark blue and green, respectively, and the plug helix in red. The amphipathic helix and TM 3 of SecE are shown in yellow at the back of SecY. Note that the model has not described the dissociation of SecA dimers known to occur upon the interaction with SecYEG (Or et al., 2002).

SecA bound: based on the structure of SecYEG-A (3DIN) (Zimmer et al., 2008). Monomeric SecA (red) has bound to SecYEG, causing a widening of the LG and a tilting in the SecE amphipathic helix. In this state the SS of the pre-protein (magenta) is well positioned to associate at the binding site at the SecY LG.

Unlocked: based on the SS-bound SecYEG structure (Hizlan et al., 2012) and the analyses described here. The SS has bound to the LG of SecY, and caused a straightening of TM 7 and a release of the SecY plug. SS binding results in conformational changes in the C4 and C5 loops and the SecE amphipathic helix that could favor the subsequent association with SecA, which is yet to fully engage. The coloured arrows represent the conformational changes described either here or in Hizlan et al. (2012), and are coloured as per the region of the complex that they relate to. Note that it is not clear in which order SecA and SS binding occur.

Open: from either an unlocked or SecA-bound state the channel is then fully primed for ATP-driven protein translocation. The structure of this open state may be similar to the recent structure of the Sec61 complex engaged with a nascent pre-secretory substrate (Voorhees and Hegde, 2016), except that in this case the pre-protein is presented by SecA rather than the ribosome.

and size-exclusion chromatography. $SecY_{7-10}EG$-containing membranes were prepared according to the standard procedure and then either oxidized with 1 mM Cu-phenanthroline or reduced with 1 mM TCEP for 1 hr at 4°C, before reverting to the standard protocol. In the case of the reduced samples, 0.5 mM TCEP was added to all buffers downstream of the reduction step.

Reconstitution into PL was carried out as previously described (Gold et al., 2007; Schulze et al., 2014), using *E. coli* polar lipids and removing excess detergent with Bio-Beads (Bio-Rad) by dialysis. Note that the presence of the lipids does not alter the oxidation state of $SecY_{7-10}EG$ (Figure S4).

Size-Exclusion Chromatography

SecYEG samples or protein standards of equal concentrations were run at a constant flow of 0.5 ml/min through a Superose 6 10/300 column in 20 mM Tris (pH 8), 130 mM NaCl, 10% glycerol, and 0.02% DDM, while monitoring the absorbance at 280 nm.

Biochemical Assays

Limited proteolysis was performed by mixing SecYEG in detergent with porcine trypsin at 0.75 μg/ml or 0.075 μg/ml, incubating at room temperature for 20 min, then mixing with lithium dodecyl sulphate gel loading buffer to quench the reaction. Results were analyzed with SDS-PAGE.

The concentration of free thiols in purified SecYEG samples was measured by assaying for binding to a thiol-reactive fluorescence dye, CPM (7-diethylamino-3-(4′-maleimidylphenyl)-4-methylcoumarin). Assays were performed using 1 μM SecYEG in detergent incubated with 10 μM CPM. CPM was excited at 389 nm and emission scans were read at 420–520 nm on a Jobin Yvon Fluorolog (Horiba Scientific).

In vitro ATPase and translocation assays involving the model substrate proOmpA (pOA) were performed essentially as described previously (Gold et al., 2007; Robson et al., 2009). In brief, the ATP turnover rate of 0.3 μM SecA was determined alone, with 2.4 μM SecYEG in PL and in the presence of 0.7 μM pOA. Reactions were followed by coupling ATP hydrolysis to NAD^+ reduction via pyruvate kinase and lactate dehydrogenase, using absorbance at 340 nm. After 30 min, untranslocated material was digested with protease K and translocation yields were determined by western blotting against pOA. Translocation bands were quantified using an Odyssey imaging system (LICOR).

SecA* was produced by labeling $SecA_{795}C$ with 5-iodoacetamidofluorescein as described previously (Deville et al., 2011). SecA* affinity assays were performed by titrating SecYEG into 10 nM SecA* in 20 mM Tris (pH 8), 130 mM NaCl, 10% glycerol, 0.02% DDM, 2 mM $MgCl_2$ and 1 mM AMP-PNP. Fluorescence readings were taken at 522 nm using a Nanodrop 3300 Fluorospectrometer (Thermo Scientific) excited with a blue light-emitting diode. The fluorescence quenching was then fitted to a tight binding equation

$$F = B_{max}\frac{E_0 + s + K_D - \sqrt{(E_0 + s + K_D)^2 - 4E_0 s}}{2E_0},$$

where F is the fluorescence signal, B_{max} is the amplitude of the fluorescence change, E_0 is the fixed concentration of SecA*, s is the binding partner (SecY variant) concentration, and K_D is the dissociation constant. Data were fitted with Prism (GraphPad).

In vitro translocation of SecYEG in bR-containing PL in the presence of the PMF was performed as described previously (Schulze et al., 2014). In vitro transcription/translation and co-translational insertion assays were performed using subunit a of *E. coli* F_1F_O-ATP synthase, F_O(a), as described previously (Schulze et al., 2014).

Tryptophan fluorescence experiments were performed with 5 μM SecYEG in 20 mM Tris (pH 8), 130 mM NaCl, 10% glycerol, and 0.02% DDM in a Hellma ultra Micro 100-μl fluorescence cuvette (Sigma-Aldrich). Fluorescence was recorded on a Jobin Yvon Fluorolog fluorometer (Horiba Scientific). The samples

were excited at 288 nm, and an emission scan was recorded between 320 and 370 nm. SDS or β-octyl glucoside was titrated into each sample at a concentration range of 0.01%–3%. Three separate emission scans were run for each concentration, and ratios of the fluorescence at 330 and 350 nm were plotted as a function of SDS or β-octyl glucoside concentration.

Molecular Dynamics Simulations

Models for the simulations were built using the crystal structures *M. jannaschii* SecYEβ (1RHZ; van den Berg et al., 2004) and *T. thermophilus* SecYE (2ZJS; Tsukazaki et al., 2008). The missing loops in SecY were added using MODELLER (Fiser et al., 2000) and amino acid substitutions were made using SCWRL4 (Krivov et al., 2009). Simulations were run in Gromacs 4.6.4 (Berendsen et al., 1995) using the OPLS all-atom force field (Jorgensen et al., 1996). The protein was embedded in a 512 united-atom palmitoyl-oleoyl phosphatidylcholine membrane (Ulmschneider and Ulmschneider, 2009) using g_membed (Wolf et al., 2010). The protein-membrane structures were solvated with explicit (Simple Point Charge) water and sodium and chloride ions to a neutral charge and concentration of 0.15 M. The systems were energy minimized using the steepest descents method, and equilibrated for 1 ns using the NPT (constant temperature and constant pressure) ensemble at 300 K with the Bussi-Donadio-Parrinello thermostat and semi-isotropic Parrinello-Rahman pressure coupling. Bond lengths were constrained using the LINCS method. Non-bonded interactions were dealt with by the Verlet cutoff, and the neighbor search list was updated every 20 steps. Long-range electrostatic interactions were calculated using the particle mesh Ewald method, and a cutoff of 1.0 nm was applied for van der Waals and short-range electrostatic interactions.

Secondary structure analyses of the loops involved residues 229–252 (C4) and 351–362 (C5) (*M. jannaschii* numbering) and used the DSSP program (Joosten et al., 2011). Distance analyses were done using the Gromacs utility g_dist.

Homology Modeling

For illustrative purposes only (Figure 4B), a model was built for *E. coli* SecYEG using MODELLER (Sali and Blundell, 1993), with 1RHZ as a template (van den Berg et al., 2004). This model was only used to demonstrate the positioning of the tryptophan residues in the *E. coli* system and was not energy minimized or subjected to MD simulation.

All molecular models are represented using PyMOL (Schrödinger).

SUPPLEMENTAL INFORMATION

Supplemental Information includes seven figures and can be found with this article online at http://dx.doi.org/10.1016/j.str.2016.02.001.

AUTHOR CONTRIBUTIONS

Experiments were designed by R.A.C., W.J.A., A.R., and I.C. Experiments were performed by R.A.C., W.J.A., J.K., A.R., S.M., S.M., and I.C. Data were analyzed by R.A.C., W.J.A., J.K., and I.C. The manuscript was written by R.A.C., W.J.A., and I.C.

ACKNOWLEDGMENTS

This work was funded by the BBSRC: R.A.C. (BBSRC South West Bioscience Doctoral Training Partnership), I.C. and W.J.A. (BB/I008675/1), and J.K. (BBSRC doctoral training grant). We are very grateful to our dedicated technical support team Jeannette Hobbs, Kathleen Moreton, and Deborah Carter. Thanks also to R. B. Sessions for continued guidance and advice. This work was carried out using the computational facilities of the Advanced Computing Research Center, University of Bristol (http://www.bris.ac.uk/acrc/). Additional simulations were carried out using computer time on ARCHER provided by HECBioSim, the UK High End Computing Consortium for Biomolecular Simulation (hecbiosim.ac.uk), supported by EPSRC.

Received: November 27, 2015
Revised: January 26, 2016
Accepted: February 5, 2016
Published: March 10, 2016

REFERENCES

Alami, M., Dalal, K., Lelj-Garolla, B., Sligar, S.G., and Duong, F. (2007). Nanodiscs unravel the interaction between the SecYEG channel and its cytosolic partner SecA. EMBO J. *26*, 1995–2004.

Arkowitz, R.A., Joly, J.C., and Wickner, W. (1993). Translocation can drive the unfolding of a preprotein domain. EMBO J. *12*, 243–253.

Auer, J., Spicker, G., and Böck, A. (1991). Presence of a gene in the archaebacterium *Methanococcus vannielii* homologous to secY of eubacteria. Biochimie *73*, 683–688.

Beck, K., Wu, L.F., Brunner, J., and Muller, M. (2000). Discrimination between SRP- and SecA/SecB-dependent substrates involves selective recognition of nascent chains by SRP and trigger factor. EMBO J. *19*, 134–143.

Berendsen, H.J.C., van der Spoel, D., and van Drunen, R. (1995). GROMACS: a message-passing parallel molecular dynamics implementation. Computer Phys. Commun. *91*, 43–56.

Bostina, M., Mohsin, B., Kühlbrandt, W., and Collinson, I. (2005). Atomic model of the *E. coli* membrane-bound protein translocation complex SecYEG. J. Mol. Biol. *352*, 1035–1043.

Briggs, M.S., Cornell, D.G., Dluhy, R.A., and Gierasch, L.M. (1986). Conformations of signal peptides induced by lipids suggest initial steps in protein export. Science *233*, 206–208.

Brodsky, J.L., Goeckeler, J., and Schekman, R. (1995). Bip and Sec63p are required for both co- and posttranslational protein translocation into the yeast endoplasmic-reticulum. Proc. Natl. Acad. Sci. USA *92*, 9643–9646.

Brundage, L., Hendrick, J.P., Schiebel, E., Driessen, A.J.M., and Wickner, W. (1990). The purified E. coli integral membrane protein SecYE is sufficient for reconstitution of SecA-dependent precursor protein translocation. Cell *62*, 649–657.

Collinson, I., Breyton, C., Duong, F., Tziatzios, C., Schubert, D., Or, E., Rapoport, T., and Kuhlbrandt, W. (2001). Projection structure and oligomeric properties of a bacterial core protein translocase. EMBO J. *20*, 2462–2471.

Collinson, I., Corey, R.A., and Allen, W.J. (2015). Channel crossing: how are proteins shipped across the bacterial plasma membrane? Philos. Trans. R. Soc. Lond. B Biol. Sci. *370*, http://dx.doi.org/10.1098/rstb.2015.0025.

de Keyzer, J., van der Does, C., Swaving, J., and Driessen, A.J. (2002). The F286Y mutation of PrlA4 tempers the signal sequence suppressor phenotype by reducing the SecA binding affinity. FEBS Lett. *510*, 17–21.

de Keyzer, J., Regeling, A., and Driessen, A.J.M. (2007). Arginine 357 of SecY is needed for SecA-dependent initiation of preprotein translocation. FEBS Lett. *581*, 1859–1864.

Derman, A.I., Puziss, J.W., Bassford, P.J., and Beckwith, J. (1993). A signal sequence is not required for protein export in prlA mutants of *Escherichia coli*. EMBO J. *12*, 10.

Deville, K., Gold, V.A., Robson, A., Whitehouse, S., Sessions, R.B., Baldwin, S.A., Radford, S.E., and Collinson, I. (2011). The oligomeric state and arrangement of the active bacterial translocon. J. Biol. Chem. *286*, 4659–4669.

Duong, F., and Wickner, W. (1997). The SecDFyajC domain of preprotein translocase controls preprotein movement by regulating SecA membrane cycling. EMBO J. *16*, 4871–4879.

Duong, F., and Wickner, W. (1999). The PrlA and PrlG phenotypes are caused by a loosened association among the translocase SecYEG subunits. EMBO J. *18*, 3263–3270.

Emr, S.D., Hanley-Way, S., and Silhavy, T.J. (1981). Suppressor mutations that restore export of a protein with a defective signal sequence. Cell *23*, 79–88.

Enno, H., Thomas, S., Siegfried, P., Dirk, G.R., Stefan, J., and Tom, A.R. (1994). Evolutionary conservation of components of the protein translocation complex. Nature *367*, 654–657.

Fiser, A., Do, R.K., and Sali, A. (2000). Modeling of loops in protein structures. Protein Sci. *9*, 1753–1773.

Flower, A.M., Osborne, R.S., and Silhavy, T.J. (1995). The allele-specific synthetic lethality of prlA-prlG double mutants predicts interactive domains of SecY and SecE. EMBO J. *14*, 884–893.

Gold, V.A.M., Robson, A., Clarke, A.R., and Collinson, I. (2007). Allosteric regulation of SecA. J. Biol. Chem. *282*, 17424–17432.

Gold, V.A.M., Robson, A., Bao, H., Romantsov, T., Duong, F., and Collinson, I. (2010). The action of cardiolipin on the bacterial translocon. Proc. Natl. Acad. Sci. USA *107*, 10044–10049.

Gold, V.A., Whitehouse, S., Robson, A., and Collinson, I. (2013). The dynamic action of SecA during the initiation of protein translocation. Biochem. J. *449*, 695–705.

Gouridis, G., Karamanou, S., Gelis, I., Kalodimos, C.G., and Economou, A. (2009). Signal peptides are allosteric activators of the protein translocase. Nature *462*, 363–367.

Hartl, F.-U., Lecker, S., Schiebel, E., Hendrick, J.P., and Wickner, W. (1990). The binding cascade of SecB to SecA to SecYE mediates preprotein targeting to the E. coli plasma membrane. Cell *63*, 269–279.

Hizlan, D., Robson, A., Whitehouse, S., Gold, V.A., Vonck, J., Mills, D., Kühlbrandt, W., and Collinson, I. (2012). Structure of the SecY complex unlocked by a preprotein mimic. Cell Rep. *1*, 21–28.

Joosten, R.P., te Beek, T.A., Krieger, E., Hekkelman, M.L., Hooft, R.W., Schneider, R., Sander, C., and Vriend, G. (2011). A series of PDB related databases for everyday needs. Nucleic Acids Res. *39*, D411–D419.

Jorgensen, W.L., Maxwell, D.S., and Tirado-Rives, J. (1996). Development and testing of the OPLS all-atom force field on conformational energetics and properties of organic liquids. J. Am. Chem. Soc. *118*, 11225–11236.

Kaufmann, A., Manting, E.H., Veenendaal, A.K.J., Driessen, A.J.M., and van der Does, C. (1999). Cysteine-directed cross-linking demonstrates that helix 3 of SecE is close to helix 2 of SecY and helix 3 of a neighboring SecE. Biochemistry *38*, 9115–9125.

Krivov, G.G., Shapovalov, M.V., and Dunbrack, R.L., Jr. (2009). Improved prediction of protein side-chain conformations with SCWRL4. Proteins *77*, 778–795.

Lill, R., Dowhan, W., and Wickner, W. (1990). The ATPase activity of SecA is regulated by acidic phospholipids, SecY, and the leader and mature domains of precursor proteins. Cell *60*, 271–280.

Mcknight, C.J., Stradley, S.J., Jones, J.D., and Gierasch, L.M. (1991). Conformational and Membrane-binding properties of a signal sequence are largely unaltered by its adjacent mature region. Proc. Natl. Acad. Sci. USA *88*, 5799–5803.

Mori, H., and Ito, K. (2001). An essential amino acid residue in the protein translocation channel revealed by targeted random mutagenesis of SecY. Proc. Natl. Acad. Sci. USA *98*, 5128–5133.

Nouwen, N., de Kruijff, B., and Tommassen, J. (1996). prlA suppressors in Escherichia coli relieve the proton electrochemical gradient dependency of translocation of wild-type precursors. Proc. Natl. Acad. Sci. USA *93*, 5953–5957.

Or, E., Navon, A., and Rapoport, T. (2002). Dissociation of the dimeric SecA ATPase during protein translocation across the bacterial membrane. EMBO J. *21*, 4470–4479.

Osborne, R.S., and Silhavy, T.J. (1993). PrlA suppressor mutations cluster in regions corresponding to three distinct topological domains. EMBO J. *12*, 3391–3398.

Plath, K., Mothes, W., Wilkinson, B.M., Stirling, C.J., and Rapoport, T.A. (1998). Signal sequence recognition in posttranslational protein transport across the yeast ER membrane. Cell *94*, 795–807.

Robson, A., Gold, V.A.M., Hodson, S., Clarke, A.R., and Collinson, I. (2009). Energy transduction in protein transport and the ATP hydrolytic cycle of SecA. Proc. Natl. Acad. Sci. USA *106*, 5111–5116.

Sali, A., and Blundell, T.L. (1993). Comparative protein modelling by satisfaction of spatial restraints. J. Mol. Biol. *234*, 779–815.

Samuelson, J.C., Chen, M., Jiang, F., Moller, I., Wiedmann, M., Kuhn, A., Phillips, G.J., and Dalbey, R.E. (2000). YidC mediates membrane protein insertion in bacteria. Nature *406*, 637–641.

Schauble, N., Lang, S., Jung, M., Cappel, S., Schorr, S., Ulucan, O., Linxweiler, J., Dudek, J., Blum, R., Helms, V., et al. (2012). BiP-mediated closing of the Sec61 channel limits Ca^{2+} leakage from the ER. EMBO J. *31*, 3784–3896.

Schulze, R.J., Komar, J., Botte, M., Allen, W.J., Whitehouse, S., Gold, V.A., Lycklama, A.N.J.A., Huard, K., Berger, I., Schaffitzel, C., and Collinson, I. (2014). Membrane protein insertion and proton-motive-force-dependent secretion through the bacterial holo-translocon SecYEG-SecDF-YajC-YidC. Proc. Natl. Acad. Sci. USA *111*, 4844–4849.

Scotti, P.A., Urbanus, M.L., Brunner, J., de Gier, J.-W.L., von Heijne, G., van der Does, C., Driessen, A.J.M., Oudega, B., and Luirink, J. (2000). YidC, the *Escherichia coli* homologue of mitochondrial Oxa1p, is a component of the Sec translocase. EMBO J. *19*, 542–549.

Smith, M.A., Clemons, W.M., Jr., Demars, C.J., and Flower, A.M. (2005). Modeling the effects of prl mutations on the *Escherichia coli* SecY complex. J. Bacteriol. *187*, 6454–6465.

Tam, P.C.K., Maillard, A.P., Chan, K.K.Y., and Duong, F. (2005). Investigating the SecY plug movement at the SecYEG translocation channel. EMBO J. *24*, 3380–3388.

Tsukazaki, T., Mori, H., Fukai, S., Ishitani, R., Mori, T., Dohmae, N., Perederina, A., Sugita, Y., Vassylyev, D.G., Ito, K., and Nureki, O. (2008). Conformational transition of Sec machinery inferred from bacterial SecYE structures. Nature *455*, 988–991.

Ulmschneider, J.P., and Ulmschneider, M.B. (2009). United atom lipid parameters for combination with the optimized potentials for liquid simulations all-atom force field. J. Chem. Theor. Comput. *5*, 1803–1813.

van den Berg, B., Clemons, W.M., Jr., Collinson, I., Modis, Y., Hartmann, E., Harrison, S.C., and Rapoport, T.A. (2004). X-ray structure of a protein-conducting channel. Nature *427*, 36–44.

van der Wolk, J.P.W., Fekkes, P., Boorsma, A., Huie, J.L., Silhavy, T.J., and Driessen, A.J.M. (1998). PrlA4 prevents the rejection of signal sequence defective preproteins by stabilizing the SecA-SecY interaction during the initiation of translocation. EMBO J. *17*, 3631–3639.

Voorhees, R.M., and Hegde, R.S. (2016). Structure of the Sec61 channel opened by a signal sequence. Science *351*, 88–91.

Wolf, M.G., Hoefling, M., Aponte-Santamaria, C., Grubmuller, H., and Groenhof, G. (2010). g_membed: efficient insertion of a membrane protein into an equilibrated lipid bilayer with minimal perturbation. J. Comput. Chem. *31*, 2169–2174.

Zimmer, J., Nam, Y., and Rapoport, T.A. (2008). Structure of a complex of the ATPase SecA and the protein-translocation channel. Nature *455*, 936–943.

Structure, Volume *24*

Supplemental Information

Unlocking the Bacterial SecY Translocon

Robin A. Corey, William J. Allen, Joanna Komar, Simonas Masiulis, Sam Menzies, Alice Robson, and Ian Collinson

Unlocking the bacterial SecY translocon

Robin A. Corey, William J. Allen, Joanna Komar, Simonas Masiulis, Sam Menzies,
Alice Robson and Ian Collinson

Supplemental information

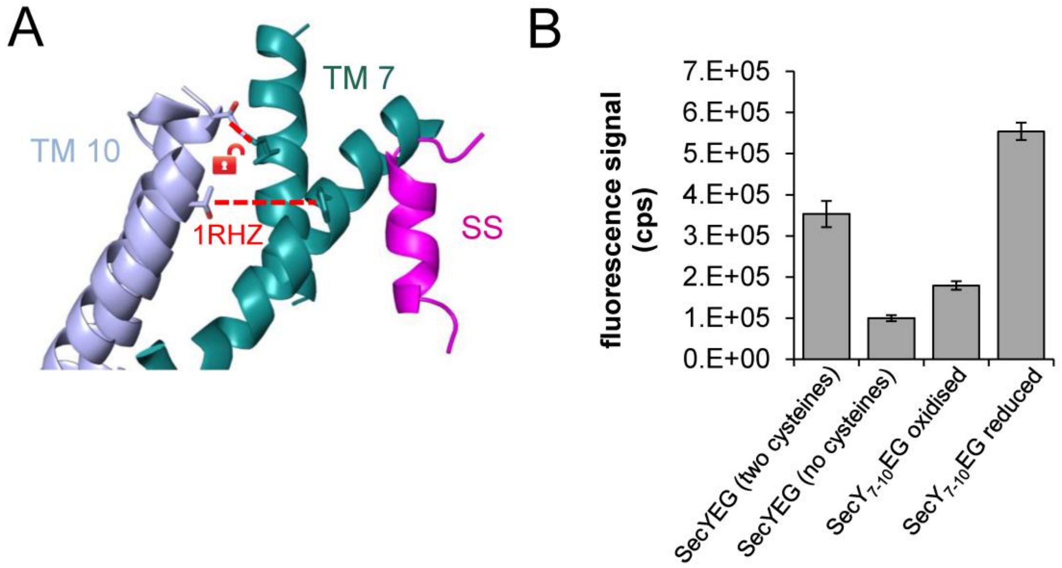

Figure S1. Related to Figure 1.

(A) Close up of SecY TMs 7 and 10 from 1RHZ (Van den Berg et al., 2004) and SS-bound SecYEG (open red padlock (Hizlan et al., 2012)) represented as cartoons and overlaid. The cysteine pair in SecY$_{7\text{-}10}$EG are shown as sticks to compare their distances (red dashed lines) in the resting state and upon SS activation respectively.

(B) Fluorescence emission following incubation of SecYEG with the CPM dye. Fluorescence is relative to number of free thiols present and is measured in counts per second (cps). Error bars are S.E.M of three repeats.

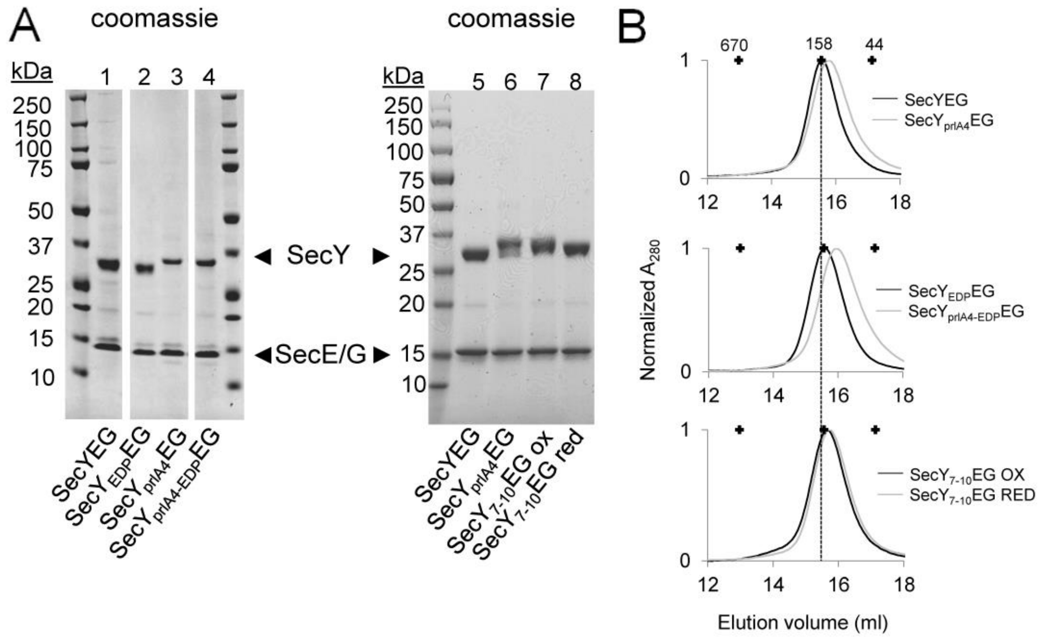

Figure S2. Related to Figure 2.

(A) SDS-PAGE analysis of the SecYEG variants stained with coomassie. The bands for SecY and SecE/G (which run at approximately the same position) and the MW markers are labelled on the side.

(B) Analytical size exclusion chromatography of the intact SecYEG complexes purified and run in detergent. Shown are the elution volumes of proteins of known molecular weight applied in order to align SecYEG complexes.

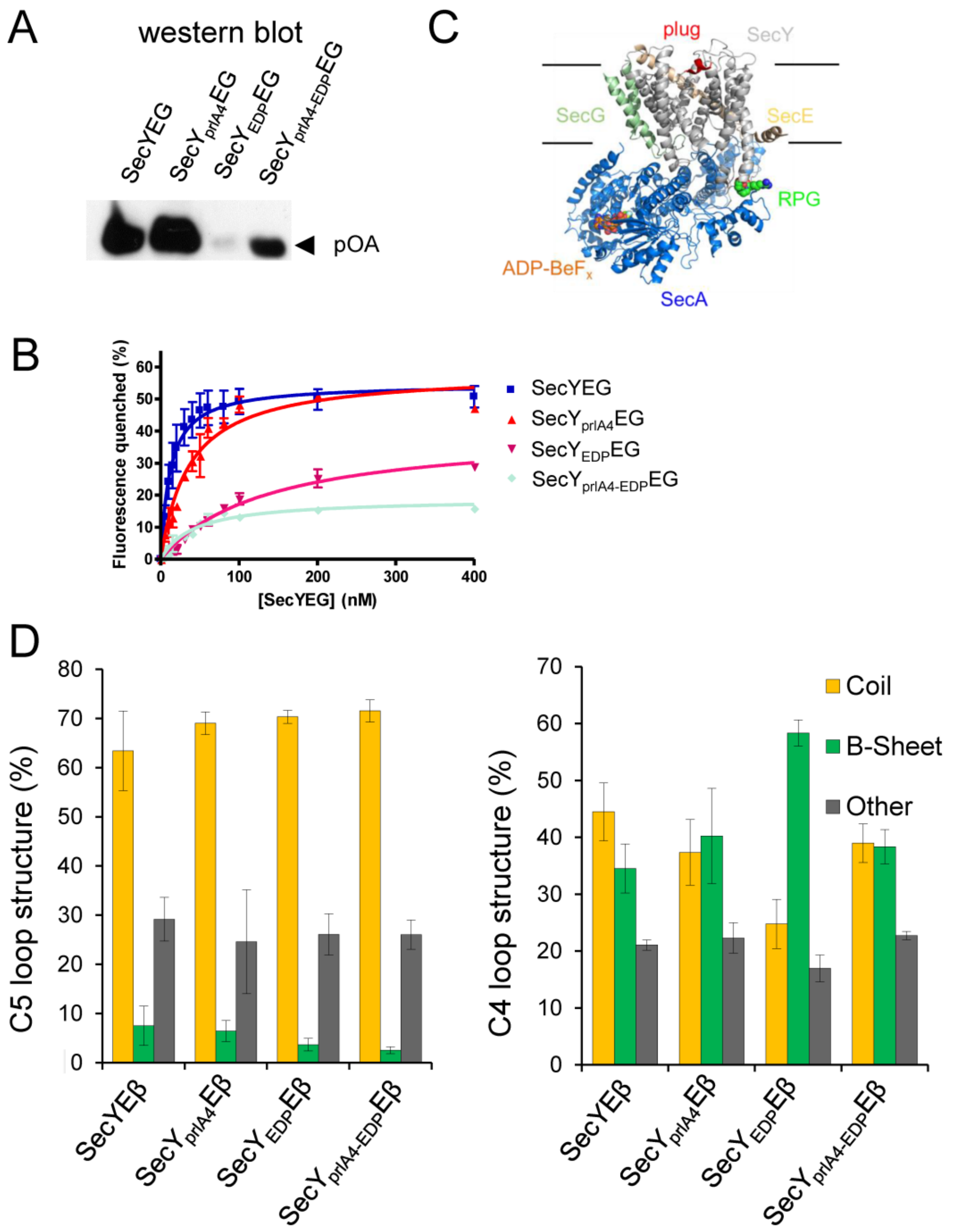

Figure S3. Related to Figure 2.

(A) Representative translocation assay for variants of SecYEG. Translocation efficiency is measured by western blotting with an antibody against a translocation substrate (pOA), and quantified as the percentage of the total pOA added to the assay. Three repeats of the assay were used for the quantification in Figure 2C.

(**B)** SecA binding assays using a fluorescently labelled SecA (SecA*). The fluorescence quench was plotted against different concentrations of SecYEG and the data was fitted to a tight binding equation. Error bars show the S.E.M of three repeats. The K_ds are plotted in Figure 2D.

(C) SecYEG-SecA (3DIN) (Zimmer et al., 2008), with the different subunits coloured and labelled accordingly. The RPG motif is shown as spheres, to illustrate its positioning with relation to SecA.

(D) MD simulations of SecYEβ mutants were analysed for secondary structure using the DSSP program (Joosten et al., 2011) on the SecY C5 loop (351-362 in *M. jannaschii* numbering) and C4 loop (residues 229-252 in *M. jannaschii* numbering). Yellow bars are unfolded regions (coil), green bars are β-sheets and grey bars are other types of secondary structure. Error bars show the S.E.M of three simulations.

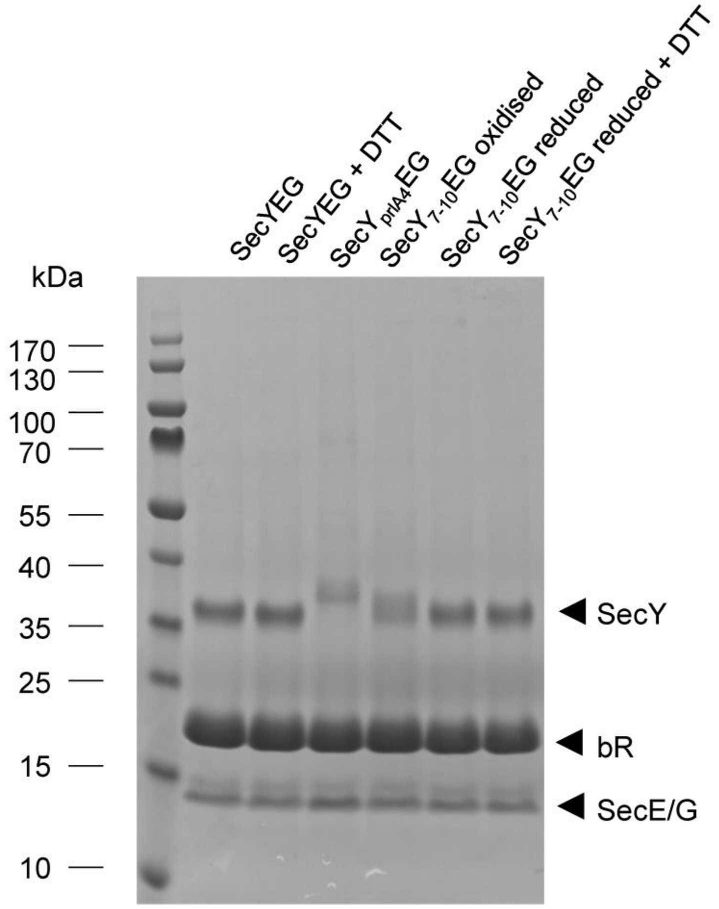

Figure S4. Related to Figure 3.

Analysis by SDS-PAGE and coomassie staining of proteo-liposomes (PL) reconstituted with SecYEG variants and bacteriorhodopsin (bR), with or without incubation with dithiothreitol (DTT). The MW markers, SecY, bR and SecE/G bands have been labelled on the side.

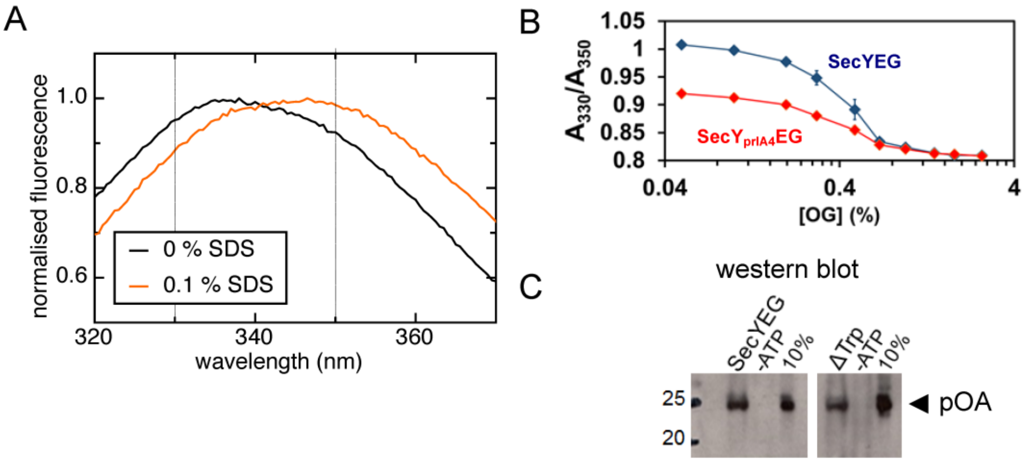

Figure S5. Related to Figure 4.

(A) Representative tryptophan fluorescence emission traces of SecYEG when excited at 288 nm, with either 0 % or 0.1 % SDS. Shown in vertical lines are the 330 and 350 nm positions used in secondary analysis. In general, SDS titration correlates with a redshift of the native SecYEG tryptophan residues, reflecting the exposure of these residues to solvent.

(B) The change in tryptophan fluorescence emission of standard SecYEG or SecY$_{prlA4}$EG in DDM, titrated with increasing concentrations of the partially destabilising detergent β-octyl glucoside. The y-axis shows the ratio of the emission amplitude at 330 and 350 nm. Note that as the x-axis is a log scale, so the 0 % β-octyl glucoside points cannot be shown. Error bars are S.E.M of three repeats.

(C) Translocation assay of PL reconstituted with SecYEG with all the native tryptophans replaced by phenylalanine. The western blot using an antibody against pOA visualises the successfully translocated substrate in the PL interior protected from protease k.

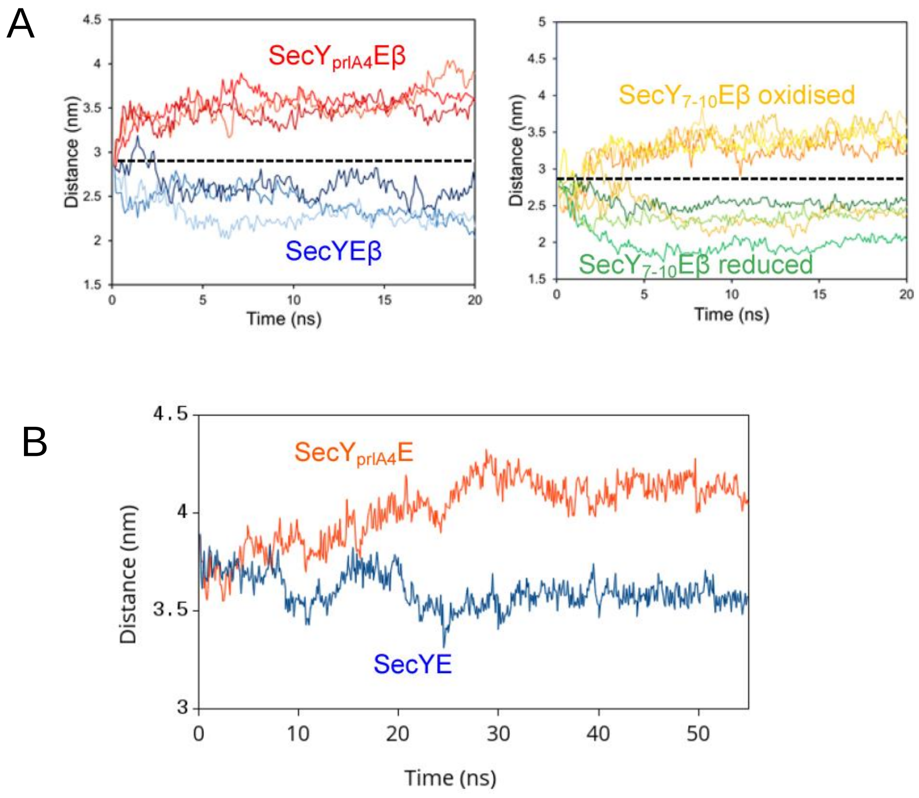

Figure S6. Related to Figure 5.

(A) Distance analysis between the SecE amphipathic helix and a rigid region of SecY, highlighted in Figure 5A; showing the first 20 ns of Figure 5B. Note that, with respect to the amphipathic helix of SecE, all of the simulations quickly diverge from the input model.

(B) Distance analysis between the SecE amphipathic helix and a rigid region of SecY (see Figure 5A) for MD simulations conducted on the gram negative *T. thermophilus* SecYE (2ZJS) (Tsukazaki et al., 2008). The standard SecYE, and a variant corresponding to the *E. coli* SecY$_{prlA4}$EG (*T. thermophilus* SecY$_{prlA4}$E) is in red. This analysis supports the data in Figure 5, and suggests that the observed shift of the SecE amphipathic helix towards the cytosol is conserved across both archaea (Figure 5) and gram-negative bacteria (shown here). Note that the region of SecE selected for distance analysis is the region containing W84, used for tryptophan fluorescence in Figure 4C.

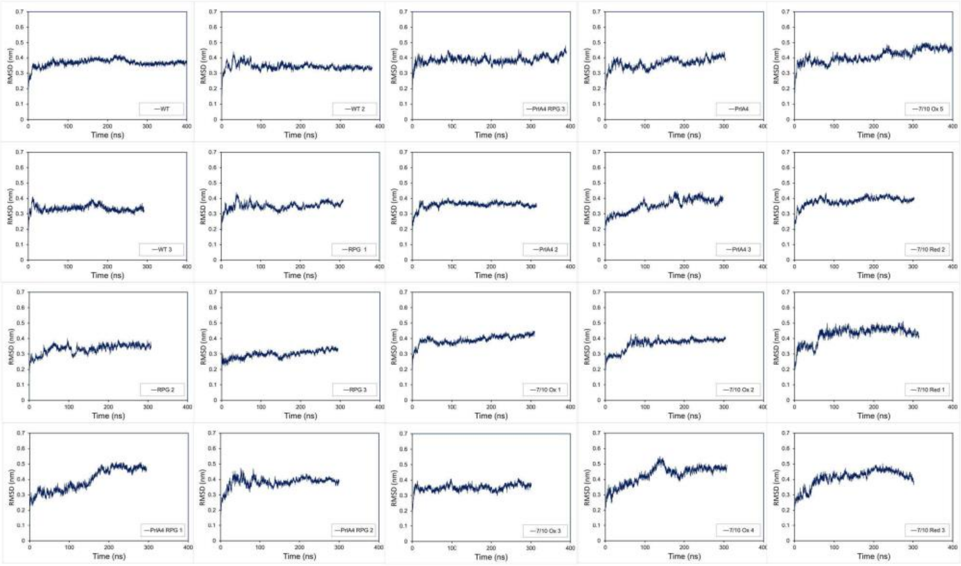

Figure S7. Related to Figure 5.

Root mean square deviation (RMSD) analysis for all of the *M. jannaschii* simulations. According to this analysis, the simulations are all stable after ~200 ns of simulation time.
